# Drug–drug interactions involving classic psychedelics: A systematic review

**DOI:** 10.1177/02698811231211219

**Published:** 2023-11-20

**Authors:** Andreas Halman, Geraldine Kong, Jerome Sarris, Daniel Perkins

**Affiliations:** 1 Melbourne School of Population and Global Health, University of Melbourne, Melbourne, VIC, Australia; 2 Department of Microbiology and Immunology, Peter Doherty Institute, University of Melbourne, Melbourne, VIC, Australia; 3 NICM Health Research Institute, Western Sydney University, Sydney, Australia; 4 Florey Institute for Neuroscience and Mental Health, University of Melbourne, Melbourne, VIC, Australia; 5 Psychae Institute, Melbourne, VIC, Australia

**Keywords:** Hallucinogens, LSD, psilocybin, mescaline, 5-MeO-DMT, DMT, ayahuasca, drug interactions, psychopharmacology, pharmacodynamics, pharmacokinetics

## Abstract

Classic psychedelics, including lysergic acid diethylamide (LSD), psilocybin, mescaline, N,N-dimethyltryptamine (DMT) and 5-methoxy-N,N-dimethyltryptamine (5-MeO-DMT), are potent psychoactive substances that have been studied for their physiological and psychological effects. However, our understanding of the potential interactions and outcomes when using these substances in combination with other drugs is limited. This systematic review aims to provide a comprehensive overview of the current research on drug–drug interactions between classic psychedelics and other drugs in humans. We conducted a thorough literature search using multiple databases, including PubMed, PsycINFO, Web of Science and other sources to supplement our search for relevant studies. A total of 7102 records were screened, and studies involving human data describing potential interactions (as well as the lack thereof) between classic psychedelics and other drugs were included. In total, we identified 52 studies from 36 reports published before September 2, 2023, encompassing 32 studies on LSD, 10 on psilocybin, 4 on mescaline, 3 on DMT, 2 on 5-MeO-DMT and 1 on ayahuasca. These studies provide insights into the interactions between classic psychedelics and a range of drugs, including antidepressants, antipsychotics, anxiolytics, mood stabilisers, recreational drugs and others. The findings revealed various effects when psychedelics were combined with other drugs, including both attenuated and potentiated effects, as well as instances where no changes were observed. Except for a few case reports, no serious adverse drug events were described in the included studies. An in-depth discussion of the results is presented, along with an exploration of the potential molecular pathways that underlie the observed effects.

## Introduction

Classic psychedelics include lysergic acid diethylamide (LSD), psilocybin (psilocin as an active agent), mescaline, N,N-dimethyltryptamine (DMT) and 5-methoxy-N,N-dimethyltryptamine (5-MeO-DMT) (Calvey and Howells, 2018). LSD has a high affinity for several serotonin (5-HT) receptors (such as 5-HT_1A/B_ and 5-HT_2A_) and also has an affinity to dopaminergic D_1–5_ receptors (Halberstadt and Geyer, 2011). The affinity for 5-HT_2A_ is important as the main mechanism behind the behavioural and psychological effects of LSD and other psychedelics is thought to be mediated through the activation of 5-HT_2A_ receptors in cortical and subcortical structures (Vollenweider and Smallridge, 2022). LSD is metabolised by cytochrome P450 (CYP) enzymes such as CYP1A2, CYP3A4, CYP2C9, CYP2C19 and CYP2D6, with particular emphasis on the primary contribution of the former two (Wagmann et al., 2019).

While LSD is chemically synthesised, psilocybin is a naturally occurring psychedelic compound found in various genera of mushrooms, including *Psilocybe, Panaeolus*, *Conocybe*, *Gymnopilus, Stropharia, Pluteus* and *Panaeolina* (Dinis-Oliveira et al., 2019). Once ingested, the body metabolises psilocybin to psilocin, which is the primary psychoactive compound (Dinis-Oliveira, 2017). The metabolism of psilocybin involves several enzymes, including aldehyde dehydrogenase, monoamine oxidase (MAO) and UDP-glucuronosyltransferase (UGT) enzymes such as UGT1A9, UGT1A10, UGT1A6, UGT1A7 and UGT1A8 (Dinis-Oliveira, 2017). Similar to LSD, psilocin acts as an agonist at 5-HT_2A_ receptors in the brain (Rickli et al., 2016), where it exerts its psychological effects (F. X. Vollenweider et al., 1998).

Mescaline (3,4,5-trimethoxyphenethylamine) is a psychedelic compound found in the North American peyote cactus (*Lophophora williamsii*), the South American San Pedro cactus (*Echinopsis pachanoi*), as well as other cacti such as the Peruvian torch cactus (*Echinopsis peruviana*), Bolivian torch cactus (*Echinopsis lageniformis*) and the leaf cactus (*Pereskia aculeata*) (Dinis-Oliveira et al., 2019). It has a high affinity for 5-HT_1A_ and 5-HT_2A/C_ receptors but is less potent than LSD, psilocin and DMT (Rickli et al., 2016). Mescaline is metabolised in the liver and broken down into several inactive compounds, with oxidative deamination occurring via MAO or diamine oxidase (Dinis-Oliveira et al., 2019).

DMT is a naturally occurring psychoactive compound found in several plants and is also endogenously produced in mammals, including humans (Jiménez and Bouso, 2022). Recreationally, DMT is consumed either in a pure form (mostly smoked) or as a key ingredient in an orally active brew called ayahuasca (Cakic et al., 2010). Ayahuasca is made by mixing a DMT-containing plant with a vine containing β-carboline alkaloids (Brito-da-Costa et al., 2020). Although the exact plant combinations can vary, a frequent mixture is made of *Psychotria viridis* (source of DMT) and *Banisteriopsis caapi* vine, whose stem and bark contain β-carbolines harmine and harmaline (Brito-da-Costa et al., 2020).

DMT is a partial agonist primarily of the 5-HT_1A_, 5-HT_2A_ and 5-HT_2C_ receptors (Carbonaro and Gatch, 2016). Oral consumption of DMT does not produce psychotropic effects due to rapid metabolism by MAO enzymes (Riba et al., 2015). However, when consumed orally as part of the ayahuasca brew, DMT becomes bioavailable due to the MAO-A inhibiting effects of harmine and harmaline, which protect DMT from deamination in the gut (Brito-da-Costa et al., 2020). DMT alone has a short half-life of 9–12 min and is rapidly metabolised by MAO-A, while CYP2D6 and, to a lesser extent, CYP2C19 are also involved in its metabolism (Good et al., 2022).

Finally, 5-MeO-DMT is a psychedelic that has been detected in numerous plant and fungal sources, as well as in the gland secretions of the *Incilius alvarius* toad (Ermakova et al., 2022). 5-MeO-DMT is a non-selective serotonin receptor agonist, exhibiting a strong affinity for 5-HT_1A/1B/1D/6/7_ but a significantly lower affinity for 5-HT_2A_ receptor subtype (Halberstadt et al., 2012; Ray, 2010). Both receptors, 5-HT_1A_ and 5-HT_2A_ activation, are involved in 5-MeO-DMT behavioural effects (Ermakova et al., 2022). 5-MeO-DMT has been observed to robustly inhibit the reuptake of serotonin but also dopamine and norepinephrine while demonstrating minimal activity in terms of releasing these three compounds (Nagai et al., 2007). The primary process by which 5-MeO-DMT is metabolised involves oxidative deamination catalysed by MAO-A, leading to the formation of 5-methoxyindoleacetic acid. In addition, a minor portion of 5-MeO-DMT undergoes O-demethylation via CYP2D6 to produce bufotenine (5-hydroxy-DMT) (Halberstadt, 2016) which is a potent 5-HT_2A_ agonist (Egan et al., 2000).

Drug–drug interactions (DDIs) can be categorised as either pharmacokinetic or pharmacodynamic interactions. Pharmacokinetic interactions occur when one drug influences the absorption, distribution, metabolism or elimination of another drug. On the other hand, pharmacodynamic interactions involve the modification of the pharmacological effect of one drug by another. These interactions can exhibit synergistic, additive or antagonistic characteristics. Additivity refers to the overall effect of a drug combination which is the sum of the effects of each individual drug, while synergy occurs when the combined effect of the drugs is greater than additive. Antagonism arises when the combined effect is less than additive (Niu et al., 2019).

One common mechanism of pharmacodynamic drug interaction is competition at the receptor level. When two drugs interact with the same receptor, they can compete for binding, leading to alterations in their pharmacological effects (Lambert, 2004). For instance, blocking the receptors where LSD, psilocin, mescaline or DMT exert their effects, such as 5-HT_2A_, could impede their psychological effects.

An example of a pharmacokinetic DDI is the inhibition of drug-metabolising enzymes, such as cytochrome P450, which are responsible for metabolising a broad range of drugs (Zhao et al., 2021). Inhibition of these enzymes by concomitant drugs or circulating metabolites can lead to altered drug metabolism and impact the drug’s effects and influence treatment outcomes (Zhao et al., 2021). Additionally, there is the potential for interaction with P-glycoprotein (P-gp), a membrane transporter that facilitates the efflux of various drugs and is present in the kidneys, liver, gastrointestinal tract and blood–brain barrier (Amin, 2013). Similar to CYP enzymes, reducing the activity of P-gp can increase the concentration of its substrates in the blood, whereas increasing its activity can decrease the concentration, leading to inadequate therapeutic effects (Amin, 2013).

For instance, CYP enzymes have a known role in LSD metabolism (Luethi et al., 2019) and therefore can affect LSD’s effects (Straumann et al., 2023; Vizeli et al., 2021). DDIs can occur even when drugs are not taken concurrently, allowing for days or even weeks between their administration. Some drugs, like fluoxetine, have prolonged inhibitory effects on CYP activity that may persist for several weeks following its discontinuation due to the extended half-life of fluoxetine and its metabolite norfluoxetine (Hemeryck and Belpaire, 2002).

Currently, there is limited literature available on the DDIs between classic psychedelics and other drugs. While two review articles have been published on this subject, the first one was limited in scope (Wyatt et al., 1976) and the second one focused solely on 3,4-methylenedioxymethamphetamine (MDMA) and psilocybin interactions with psychiatric medications (Sarparast et al., 2022). To fill this gap, this systematic review aimed to provide a comprehensive overview of the current state of research on DDIs between classic psychedelics and any other drugs. We conducted a thorough literature search and reported on both physiological and subjective outcomes. This review offers valuable insights into the potential risks and benefits of combining classic psychedelics (LSD, psilocybin, mescaline, DMT and 5-MeO-DMT) with other drugs, thereby guiding researchers and clinicians in this field.

## Methods

This review was registered in PROSPERO (CRD42022336092) and followed the latest Preferred Reporting Items for Systematic Reviews and Meta-Analyses (PRISMA) (2020) guidelines (Page et al., 2021). A keyword search for articles pertaining to classical psychedelics (LSD, psilocybin, mescaline, DMT and ayahuasca) was initially conducted on June 5, 2022, in three primary scientific databases: PubMed, PsycINFO and Web of Science (with no year restriction). These databases comprise a general journal articles database and a database specialised in biomedicine and psychology research. Search terms included keywords (including synonyms) related to classic psychedelics in the title, abstract, keywords, full text (where available) and MeSH terms, as well as keywords related to drug interactions, side effects and adverse reactions. The search was not limited by the time period to capture all relevant articles. A total of 2151 articles were identified during the first search. The search was repeated on September 2, 2023, resulting in the identification of 262 additional articles published since the initial search. The full search terms for each database are provided in the Supplemental Material S1. Furthermore, on September 2, 2023, an additional search was carried out using the same search parameters to identify articles detailing drug interactions involving 5-MeO-DMT (full search terms are provided in Supplemental Material S2). A total of 507 records relating to 5-MeO-DMT were found.

Additionally, to ensure comprehensive coverage, a search was conducted in the Multidisciplinary Association for Psychedelic Studies (MAPS) comprehensive online ‘Psychedelic Bibliography’ database (https://bibliography.maps.org), which contains scientific but also non-scientific articles specifically about psychedelics, dating back to 1841. At the time of the search (November 20, 2022), the database contained 13,237 records. All records were downloaded, followed by keyword searches in all titles and abstracts (targeting ‘LSD’, ‘psilocybin’, ‘mescaline’, ‘DMT’ and ‘ayahuasca’, including synonyms and variations), resulting in a total of 6336 records.

Furthermore, we manually searched a registry of clinical trials at ClinicalTrials.gov on April 20, 2023, to find any articles containing results from clinical trials that were missed in the database search and conducted reference lists checking of included studies to find additional missing articles. An additional search of ClinicalTrials.gov was conducted on September 2, 2023 to find clinical trials involving 5-MeO-DMT.

**Figure 1.**
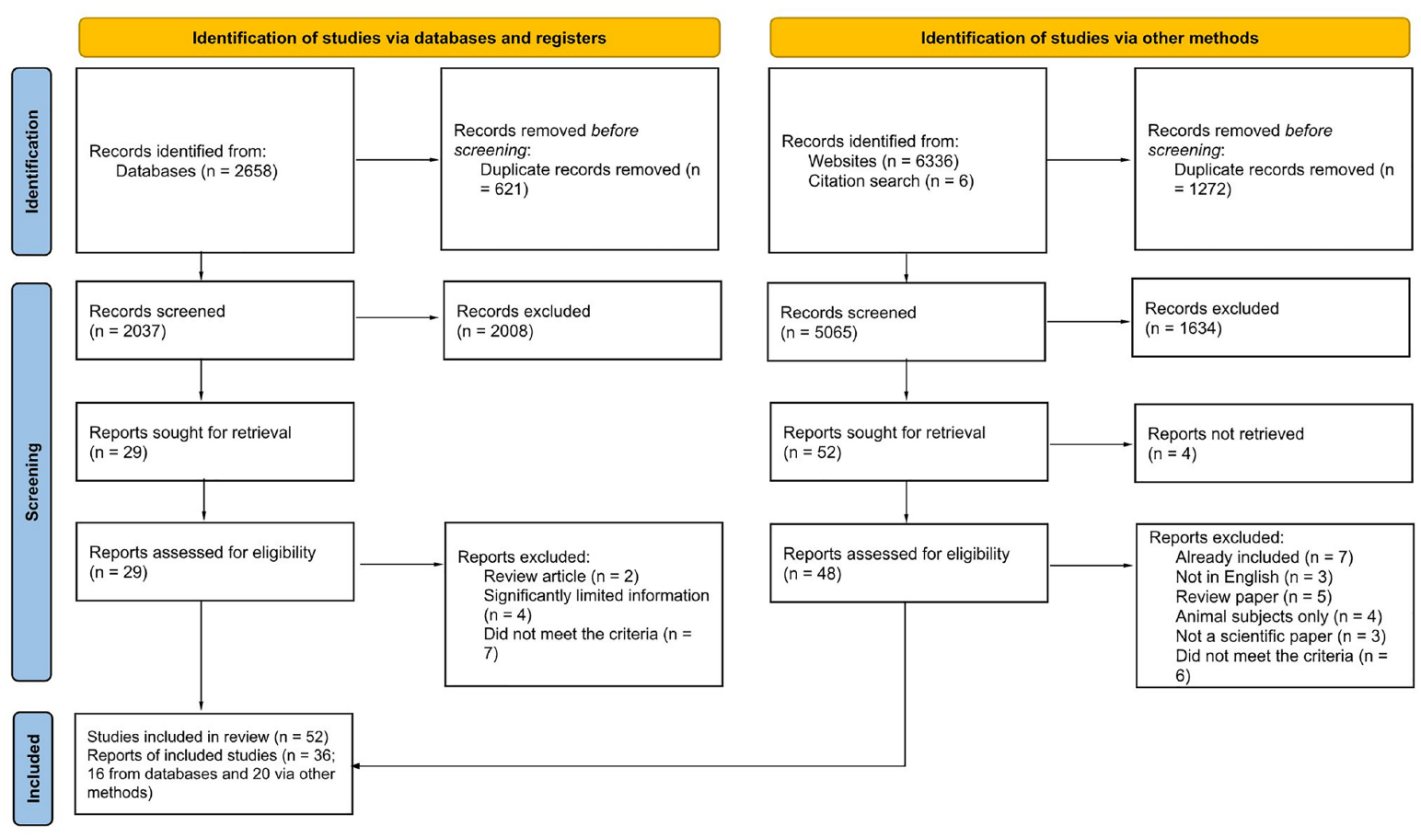
PRISMA flow chart depicting the identification of studies in the systematic review process. PRISMA: Preferred Reporting Items for Systematic Reviews and Meta-Analyses.

Records were screened in three phases: first, those found via a scientific database search; second, those from the MAPS bibliography database and finally, records from a search for 5-MeO-DMT. In all cases, a systematic review application Catchii (Halman and Oshlack, 2023) was used. Duplicate removal was performed using Catchii’s duplicate detection method and the results were manually verified before removal. Two researchers independently conducted title and abstract screening, both of whom were blinded to each other’s decisions. The inclusion criteria were as follows: (1) any article on human participants, (2) any article describing the usage of any classical psychedelics with another drug, (3) studies describing physiological and/or psychological effects of classical psychedelics with another drug (or the lack of), (4) studies that were either randomised controlled trials, observational studies (cohort, case–control, cross-sectional) or case reports/studies, and (5) studies published in English. Disagreements between reviewers were discussed until a consensus decision was reached. Records that did not meet the population, intervention and outcome criteria were excluded. After removing duplicates, a total of 7102 records (1667 from the first phase, 5065 from the second phase and 370 from the third phase) underwent the first stage of screening, which involved title and abstract analysis. Subsequently, the full text of the 77 reports that passed this stage was assessed in the second stage, including articles identified from citation searches. Due to the small number of records retrieved from the updated scientific database search, each title and abstract was assessed directly within the search results. The summary of results is shown on the PRISMA flow diagram (Figure 1). Data from all eligible records were extracted by the authors of this review, who corroborated each other’s findings. Outcome measures included physiological and psychological outcomes.

## Results

The results of this systematic review include studies investigating DDIs between psychedelic drugs (LSD, psilocybin, mescaline, 5-MeO-DMT, DMT and ayahuasca) and other drugs. Regarding LSD, the review examined interactions with antipsychotics (chlorpromazine), mood stabilisers (lithium), various antidepressants including selective serotonin reuptake inhibitors (SSRIs; fluoxetine, sertraline, paroxetine and trazodone), tricyclic antidepressants (TCAs; imipramine, desipramine and clomipramine), as well as monoamine oxidase inhibitors (MAOIs; phenelzine, isocarboxazid, nialamide and iproniazid) and other substances (azacyclonol). Recreational substances (alcohol and MDMA), as well as other substances such as ketanserin, reserpine, niacin, scopolamine and phenoxybenzamine, were also included.

Regarding psilocybin, the review contains reports on its interactions with anxiolytics (buspirone), antipsychotics (chlorpromazine, haloperidol and risperidone), SSRI antidepressants (escitalopram) and recreational drugs (alcohol). Other investigated substances included ketanserin and ergotamine. In the case of mescaline, the review examined interactions with antipsychotics (chlorpromazine and promazine), antidepressants (azacyclonol) and the compound 2,4,5-trimethoxyphenethylamine (2C-O). For DMT, the review explored interactions with MAOI antidepressants (iproniazid), as well as other substances like racemic pindolol and methysergide. Lastly, one report of the SSRI antidepressant fluoxetine was found for ayahuasca and two case reports for 5-MeO-DMT involving the use of β-carbolines.

Results of studies included in this review are provided as separate tables for LSD (Table 1), psilocybin (Table 2), mescaline (Table 3), DMT together with ayahuasca (Table 4) and 5-MeO-DMT (Table 5), grouped by drug class. More detailed narrative descriptions of the results from each study are shown in Supplemental Material S3. Additionally, a combined table (Supplemental Table S1) summarises all the results from Tables 1 to 5, along with listing the potentially relevant molecular targets and actions of each drug.

**Table 1.** Summary of studies and case reports describing interactions (or the absence thereof) between LSD and other drugs.

Author and year	Study type	Drug	Dose	Participants	Outcome
Isbell and Logan (1957)	Placebo-controlled trial	Antipsychotics: chlorpromazine	(1) Pretreatment with 50–100 mg chlorpromazine 30 min prior to 40–60 μg LSD. (2) Administration of 75 mg chlorpromazine at the height of 60 μg LSD. (3) Administration of either 75 mg oral or 25 mg intramuscular chlorpromazine 30 min after ingestion of 60–150 μg LSD.	*N* = 6–9 (adult male drug addicts)	Pretreatment of chlorpromazine significantly reduced mental, hallucinations, visual perception distortion, anxiety and pupillary reactions to LSD. Intramuscular, but not oral, administration of chlorpromazine after ingestion of LSD significantly blocked the effects of LSD.
Murphree (1962)	Controlled trial	Antipsychotics: chlorpromazine	Pretreatment with 25 mg chlorpromazine 30 min before ingestion of 15–20 μg LSD. Co-administration of chlorpromazine with LSD.	*N* = 18 (healthy)	Pretreatment of chlorpromazine 30 min before LSD blocked LSD effects and elevated the threshold dose. When chlorpromazine was given simultaneously or after LSD ingestion, no blocking effects were observed.
Abramson et al. (1960)	Controlled trial	Antipsychotics: chlorpromazine	Three experiments of oral administration of 50 mg chlorpromazine at 1.5 h before, after or simultaneous with 50 μg of LSD.	*N* = 3 (healthy)	Participants who took chlorpromazine before LSD ingestion showed enhanced positive responses to LSD. Chlorpromazine ingestion after LSD also increased positive responses to LSD and reduced the usual anxiety symptoms such as sweating and moist palms. Simultaneous ingestion of chlorpromazine and LSD produced similar effects as LSD alone.
Schwarz (1967)	Case series	Antipsychotics: chlorpromazine	25 mg chlorpromazine was given 4 h after 100 μg of LSD (intravenous).	*N* = 2 (schizophrenic) Age: 22 and 33	Both patients reported that chlorpromazine administration increased anxiety and intensified the effects of LSD.
Bonson and Murphy (1996)	Case series	Mood stabilisers: lithium	600–1000 mg/day of lithium for 7–50 weeks prior to 200 μg of LSD.	*N* = 3 (patients treated for depression) Age: 21–29	Participants experienced earlier onset as well as increased hallucinatory and psychological effects of LSD.
Strassman (1992)	Case report	Antidepressants (SSRI): fluoxetine	20 mg/day fluoxetine; LSD dose unknown but one that produces full hallucinogenic effects.	*N* = 1 (patient for dysthymia) Age: 38	Markedly decreased sensitivity to LSD and required 2 to 3 times more than the usual dose to elicit a full hallucinogenic effect of LSD.
Bonson et al. (1996)	Cross-sectional study	Antidepressants (SSRI): fluoxetine	Pretreatment of fluoxetine of 20–80 mg/day for 2–150 weeks followed by 250 μg of LSD.	*N* = 18 (patients treated for depression)	Fluoxetine delayed the onset of LSD effects (8 out of 18 patients) and markedly diminished the hallucinogenic and psychological effects as well as the overall response of LSD regardless of the dose or duration of fluoxetine pretreatment.
Bonson et al. (1996)	Cross-sectional study	Antidepressants (SSRI): sertraline	Pretreatment of sertraline of 50–200 mg/day for 3–40 weeks followed by 200 μg of LSD.	*N* = 11 (patients treated for depression)	Sertraline did not affect the onset of LSD effects. Most of the participants experienced decreased physical, hallucinogenic and psychological effects of LSD regardless of dosage or duration of sertraline treatment.
Bonson et al. (1996)	Cross-sectional study	Antidepressants (SSRI): paroxetine	Pretreatment of 20–40 mg/day paroxetine for 3 30 weeks followed by 150 μg of LSD.	*N* = 3 (patients treated for depression)	Paroxetine pretreatment did not affect the onset of effects but attenuated the hallucinogenic as well as physiologic effects of LSD and overall, reduced responses to LSD.
Bonson et al. (1996)	Case report	Antidepressants (SARI): trazodone	Pretreatment with 200 mg/day for 24 weeks followed by a ‘moderate’ dose of LSD (no more details given).	*N* = 1 (patient treated for depression)	No changes to the onset of LSD effects but reduced the hallucinogenic and physiological effects of LSD as well as the overall response of LSD.
Bonson and Murphy (1996)	Case series	Antidepressants (TCA): imipramine	175–200 mg/day for 8–40 weeks before the LSD dose of 80–200 μg.	*N* = 2 (patients treated for depression) Age: 26–28	Patients experienced earlier onset of LSD effects and increased hallucinatory as well as psychological effects.
Bonson and Murphy (1996)	Case series	Antidepressants (TCA): desipramine	200 mg/day for 100–150+ weeks before 100–150 μg of LSD dose.	*N* = 2 (patients treated for depression) Age: 27–32	Patients experienced earlier onset of LSD effects and increased hallucinatory as well as psychological effects.
Bonson and Murphy (1996)	Case report	Antidepressants (TCA): clomipramine	125 mg/day for 12 weeks before a ‘moderate’ dose of LSD.	*N* = 1 (patient treated for alcoholism) Age: 25	The patient experienced an earlier onset of LSD effect, increased physical and hallucinatory as well as psychological effects.
Bonson and Murphy (1996)	Case series	Antidepressants (MAOI): phenelzine	60–75 mg/day for 12 weeks before 150 μg of LSD.	*N* = 2 (patients treated for depression)	No change to the onset of LSD effects but nearly abolished the subjective responses of LSD including hallucination and psychological effects
Resnick et al. (1964)	Controlled trial	Antidepressants (MAOI): isocarboxazid	Pretreatment with 30 mg/day isocarboxazid for 5 days or 2 weeks before 40 μg of oral ingestion of LSD.	*N* = 4 (healthy)	Isocarboxazid pretreatment attenuated psychological, autonomic and neurologic responses of LSD including blood pressure, heart rate, sensory and motor functions.
Grof and Dytrych (1965)	Controlled trial	Antidepressants (MAOI): nialamide	250–500 mg/day of parenteral administration of nialamide for several days, followed by 150–300 mg/day peroral application of nialamide, and then 150 μg of LSD on test day. Pretreatment of nialamide daily for 3 weeks to reach a total dose of 3500 mg, followed by 150 and 500 μg of LSD on test day.	*N* = 14 (neurotic patients)	Premedication with nialamide blocked reactions to LSD and patients were able to tolerate 150 and 500 μg of LSD without clinical or psychotic reactions/symptoms of LSD and minimal subjective complaints.
DeMaar et al. (1960)	Placebo-controlled trial	Antidepressants (MAOI): iproniazid	100 mg iproniazid with LSD in doses of 25, 50 and 100 μg.	*N* = 16 (healthy)	No change to the physical or subjective effects of LSD.
Fabing (1955)	Case series	Antidepressants: azacyclonol	(1) Pretreatment with 10–30 mg azacyclonol for 1 week before ingestion of 100 μg LSD. (2) Pretreatment of participants with a total of four doses of 50 mg azacyclonol at 50, 38, 26 and 2 h before ingestion of 100 μg LSD. (3) Premedication with azacyclonol at either 5 or 10 mg twice daily for 7 days, followed by ingestion of 100 μg LSD. Five weeks later, participants received the same dose of LSD again without azacyclonol premedication.	(1) *N* = 6 (healthy) Age: 19–40 (2) *N* = 2 (3) *N* = 2	(1) Azacyclonol pretreatment reduced the psychological effects of LSD but had no effect on the physical effects of LSD. (2) The hallucinogenic effects of LSD were abolished by pretreatment of azacyclonol 50 h before ingestion of LSD. Other neurological effects like attention deficit or physiological effects like nausea or tight sensations were still experienced by the participants (3) Premedication of azacyclonol for 7 days, followed by a 5-week washout did not block the effects of LSD. One subject was administered 40 mg azacyclonol intravenously at the fifth hour post-LSD ingestion, reported decreased anxiety and decreased psychological effects.
Clark (1956)	Placebo-controlled trial	Antidepressants: azacyclonol	(1) Pretreatment with azacyclonol for 10–40 mg for 2–5 days, followed by 100 μg LSD. (2) Intravenous injection of 100 mg azacyclonol was administered after 100 μg LSD.	*N* = 5 (schizophrenic patients)	(1) No evidence of LSD-blocking effects by azacyclonol was observed. (2) No evidence of azacyclonol effects in changing the effects of LSD.
Isbell and Logan (1957)	Placebo-controlled trial	Antidepressants: azacyclonol	Pretreatment with 20 mg of oral azacyclonol for 7 days and an additional 20 mg 2 h before administration of 60 μg LSD. An additional dose of 40 mg was given intravenously 3 h after LSD administration.	*N* = 12	No reduction of any aspect of the LSD reaction was observed from the blocking experiment. No significant diminution in any aspect of the LSD reaction could be observed after azacyclonol administration.
Barrett et al. (2000)	Cross-sectional study	Recreational: alcohol	Self-report study. Ingestion of alcohol concurrent with the administration of LSD (no more details given).	*N* = 22 Age: 18–28 years	Ingestion of LSD concurrent with alcohol consumption diminished the effects of alcohol. No differences in the subjective effects of LSD were associated with alcohol use.
Straumann et al. (2023)	Placebo-controlled trial	Recreational: MDMA	Co-administration of 100 μg LSD and 100 mg MDMA.	*N* = 24 (healthy) Age: 25–54 years	Co-administration of MDMA and LSD resulted in a longer psychedelic experience than with LSD alone (average of 1.5 h). There were no significant differences in subjective effects, as measured by Visual Analog Scales, 5 Dimensions of Altered States of Consciousness, Mystical Experience Questionnaire and Altered States of Consciousness Rating Scale, compared to LSD alone. Increased blood pressure and heart rate compared to LSD alone.
Preller et al. (2018)	Randomised, placebo-controlled trial	Other: ketanserin	Pretreatment with 40 mg ketanserin 60 min before treatment with 100 μg LSD.	*N* = 24 (healthy)	Ketanserin fully blocked the subjective and mental effects of LSD, reduced communication among brain areas involved in planning and decision-making (fMRI and BOLD).
Olbrich et al. (2021)	Randomised, placebo-controlled trial	Other: ketanserin	Pretreatment with 40 mg ketanserin 60 min before treatment with 100 μg LSD.	*N* = 17 (healthy)	Ketanserin pretreatment shifted participants on LSD towards parasympathetic activity which is associated with less intense subjective experience including less audio-visual synaesthesia, 5D-ASC elementary imagery and experience of unity.
Holze et al. (2021)	Randomised, placebo-controlled trial	Other: ketanserin	Pretreatment with 40 mg ketanserin 60 min before treatment with 200 μg LSD.	*N* = 16 (healthy)	Ketanserin pretreatment followed by a high dose of LSD at 200 μg blocked subjective drug effects, ego dissolution, anxiety and oceanic boundlessness score to levels that were similar to a 25 μg LSD dose. Physical effects such as blood pressure and body temperature induced by 200 μg LSD + ketanserin were comparable to placebo controls.
Becker et al. (2023)	Randomised, placebo-controlled trial	Other: ketanserin	Oral administration of 40 mg ketanserin 60 min after ingestion of 100 μg LSD.	*N* = 24 (healthy)	Administration of ketanserin after the effects of LSD has reversed subjective and autonomic responses to LSD in humans. Duration of LSD effects was reduced from 8.5 h to 3.5 h (~60%) by ketanserin. Ketanserin did not alter overall mystical experiences or reverse the LSD-induced increase of plasma BDNF, or alter the pharmacokinetics of LSD.
Resnick et al. (1965)	Placebo-controlled trial	Other: reserpine	Pretreatment with 0.5 mg/day reserpine for 2 weeks before ingestion of 75 μg of LSD.	*N* = 3 (healthy)	Pretreatment with reserpine enhanced the effects of LSD on autonomic and neurologic responses as well as psychological reactions to LSD.
Isbell and Logan (1957)	Placebo-controlled trial	Other: reserpine	Pretreatment with either 2.5 mg oral or 2 mg intramuscular reserpine 2, 10 and 22 h prior to administration of 60 μg of LSD.	*N* = 12 (non-psychotic drug addicts)	Reserpine intensified LSD effects of nervousness and confusion, including nasal stuffiness, nausea, diarrhoea, vomiting and lethargy in a dose-dependent manner. Participants who underwent intramuscular administration of reserpine reported intensified LSD effects.
Freedman (1963)	Controlled trial	Other: reserpine	Pretreatment with a single dose of 10 mg reserpine 2 days prior to 120 μg of LSD.	*N* = 14 (schizophrenic females)	Reserpine enhanced the physical effects of LSD including prolonged tremors and akathisia, effects were ‘unpleasant’ and lasted longer.
Agnew and Hoffer (1955)	Controlled trial	Other: niacin	Pretreatment with 3 g of oral niacin for 3 days prior to 100 μg of LSD ingestion on test day. Intravenous injection of 0.2 g niacin post-LSD ingestion (100 μg) at the height of LSD effects.	*N* = 10 (healthy males) Age: 19–31, mean of 26 years	Pretreatment with niacin delayed the onset of LSD effects and prevented most of the perceptual changes from occurring. Niacin administration post-LSD ingestion attenuated all effects of LSD within 5 min and markedly diminished the proprioceptive, perceptual, cognitive and motor effects of LSD.
Isbell et al. (1959)	Placebo-controlled trial	Other: scopolamine	Subjects were administered with either of the following four different doses of scopolamine at 0.42, 0.64, 0.85 and 1.3 mg followed by simultaneous administration of 60 μg of LSD.	*N* = 12	Scopolamine did not alter LSD-induced effects on patellar reflex, pupil size, blood pressure or subjective clinical effects.
Isbell et al. (1959)	Placebo-controlled trial	Other: phenoxybenzamine	Ten patients received 0.5, 1.0, and 1.0 mg/kg. of phenoxybenzamine hydrochloride 24, 11, and 2 hours prior to administration of 1.0 μg/kg. of LSD.	*N* = 10	None of the doses of phenoxybenzamine pretreatment did alter LSD-induced effects on pulse rate, blood pressure or subjective clinical effects.

5D-ASC: 5-dimensional altered states of consciousness; BOLD: blood-oxygen-level-dependent; fMRI: functional magnetic resonance imaging; LSD: lysergic acid diethylamide; MAOI: monoamine oxidase inhibitor; MDMA: 3,4-methylenedioxymethamphetamine; SSRI: selective serotonin reuptake inhibitor; TCA: tricyclic antidepressant.

**Table 2.** Summary of studies and case reports describing interactions (or the absence thereof) between psilocybin and other drugs.

Author and year	Study type	Drug	Dose	Participants	Outcome
Pokorny et al. (2016)	Randomised, placebo-controlled trial	Anxiolytics: buspirone	Pretreatment with 20 mg oral buspirone 60 min before oral administration of 0.17 mg/kg psilocybin.	*N* = 19 (healthy)	Buspirone pretreatment markedly reduced psilocybin-induced visual perception distortion.
Keeler (1967)	Randomised, placebo-controlled trial	Antipsychotics: chlorpromazine	Pretreatment with 50 mg chlorpromazine 2 h before 0.2 mg/kg psilocybin.	*N* = 8 (healthy)	Pretreatment with chlorpromazine significantly decreased psilocybin-induced pupil dilation and visual perception distortion.
F. X. Vollenweider et al. (1998)	Randomised, placebo-controlled trial	Antipsychotics: haloperidol	Pretreatment with intravenous 0.021 mg/kg haloperidol 75 min before oral administration of 0.25 mg/kg psilocybin.	*N* = 15 (healthy)	Haloperidol pretreatment reduced psilocybin effects on the Oceanic boundlessness, measures of derealisation and depersonalisation phenomena but increased anxiety without any effects on visual hallucinations or delayed response tasks.
F. X. Vollenweider et al. (1998)	Randomised, placebo-controlled trial	Antipsychotics: risperidone	Pretreatment with either 0.5 mg or 1 mg oral risperidone 90 min before oral administration of 0.25 mg/kg psilocybin.	*N* = 15 (healthy)	Risperidone pretreatment attenuated the effects of psilocybin in a dose-dependent manner.
Becker et al. (2022)	Randomised, placebo-controlled trial	Antidepressants (SSRI): escitalopram	Pretreatment with 10 mg/day for 7 days, followed by 20 mg/day for 7 days, and a single dose on test day 2 h before 25 mg psilocybin treatment.	*N* = 23 (healthy)	Escitolopram pretreatment reduced the negative acute effects of psilocybin (subjective bad drug effects, anxious-ego dissolution, anxiety and “nadir effects”). The positive mood and mind-altering effects of a full dose of psilocybin were not reduced by pretreatment.
F. X. Vollenweider et al. (1998)	Randomised, placebo-controlled trial	Other: ketanserin	Pretreatment with either 20 or 40 mg oral ketanserin 75 min before oral administration of 0.25 mg/kg psilocybin.	*N* = 27 (healthy) Age: mean 29.7	Ketanserin pretreatment blocked psilocybin effects in a dose-dependent manner on the delayed response task and Altered State of Consciousness Rating scale.
Carter et al. (2005)	Controlled trial	Other: ketanserin	Pretreatment with 50 mg oral ketanserin 90 min before oral administration of 0.215 mg/kg psilocybin.	*N* = 8 (healthy)	Ketanserin pretreatment blocked the subjective effects of psilocybin based on the 5D-ASC rating scale (anxious ego dissolution, euphoria, depersonalisation and hallucination). Ketanserin did not rescue psilocybin-induced impairment in attentional tracking performance.
Carter et al. (2007)	Controlled trial	Other: ketanserin	Pretreatment with 50 mg oral ketanserin 90 min before oral administration of 0.215 mg/kg psilocybin.	*N* = 10 (healthy)	Ketanserin pretreatment blocked the subjective effects on the 5D-ASC rating scale (anxious ego dissolution, depersonalisation, euphoria and hallucination) but did not influence either the psilocybin-induced slowing of binocular rivalry or negative drug symptoms (alertness and arousal).
Pokorny et al. (2016)	Randomised, placebo-controlled trial	Other: ergotamine	Pretreatment with 3 mg oral ergotamine 100 min before oral administration of 0.17 mg/kg psilocybin.	*N* = 17 (healthy)	Ergotamine did not significantly alter subjective experiences (5D-ASC scores).
Barrett et al. (2000)	Cross-sectional study	Recreational: alcohol	Non-intoxicating levels of alcohol consumed either before or after psilocybin (self-reported, doses unknown).	*N* = 15	The subjective effects of alcohol are antagonised by psilocybin, but the subjective effects of psilocybin were diminished in only 2 (out of 15) participants while only 1 reported enhanced effects, the rest (80%) reported unchanged effects.

5D-ASC: 5-dimensional altered states of consciousness; SSRI: selective serotonin reuptake inhibitor.

**Table 3.** Summary of studies and case reports describing interactions (or the absence thereof) between mescaline and other drugs.

Author and year	Study type	Drug	Dose	Participants	Outcome
Lesse (1958)	Controlled trial	Antipsychotics: chlorpromazine and promazine	Mescaline (intravenous) or LSD (oral) followed by intravenous chlorpromazine or promazine (no more details given).	*N* = 25 (patients with schizophrenia)	The administration of chlorpromazine or promazine led to a reduction in acute anxiety symptoms in 68% (17/25) of participants.
Fabing (1955)	Case series	Antidepressants: azacyclonol	(1) Pretreatment with 50 mg azacyclonol at 50, 38, 26 and 2 h before ingestion of 400 mg mescaline sulphate. 2) Placebo instead of pretreatment, at 4.5 or 5 h mark 100 mg azacyclonol (intravenous).	*N* = 4 (healthy) Age: 20–45	(1) Pretreatment with azacyclonol likely attenuated the effects of mescaline. (2) Azacyclonol treatment during the height of the experience started to block the actions of mescaline.
Clark (1956)	Case report	Antidepressants: azacyclonol	40 mg azacyclonol four times a day for 3 days before ingesting 200 mg and 400 mg mescaline sulphate.	*N* = 1 (healthy)	No differences in mescaline intoxication when ingesting it with or without azacyclonol pretreatment.
Dittrich (1971)	Case report	Other: 2C-O	Pretreatment with 100–200 mg 2C-O and 45 min later 100 mg mescaline.	*N* = 1 (healthy) Age: 29	Pretreatment with 2C-O potentiated the effects of mescaline as the ability to concentrate decreased and the time taking a test increased.

LSD: lysergic acid diethylamide; 2C-O: 2,4,5-trimethoxyphenethylamine.

**Table 4.** Summary of studies and case reports describing interactions (or the absence thereof) between DMT, ayahuasca and other drugs.

Author and year	Study type	Drug	Dose	Participants	Outcome
Sai Halasz (1963)	Controlled trial	Antidepressants (MAOI): iproniazid	Pretreated with iproniazid (4 days 100 mg/daily) followed by a pause of 2 days. On the following day: five volunteers received a dose of 0.65–0.83 mg/kg DMT and two individuals a lower dose of 0.35–0.55 mg/kg DMT.	*N* = 7 (healthy) Age: 21–36 years 4 males/3 females	Volunteers who received the lower dose of DMT did not experience hallucinations or disruptions in time and space orientation but reported an ‘odd’ feeling. The five receiving the higher dose experienced less intense DMT effects lasting for 14–24 min.
Strassman (1996)	Randomised, placebo-controlled trial	Other: racemic pindolol	Racemic pindolol (30 mg oral) and 0.1 mg/kg DMT (intravenous)	*N* = 12 (healthy)	Pindolol pre-treatment enhanced DMT effects by 2–3 times. Significant enhancement in 4–6 clinical clusters in the hallucinogen rating scale. Mean arterial blood pressure effects were enhanced, heart rate responses were blunted, prolactin responses were reduced.
Sai-Halasz (1962)	Controlled trial	Other: methysergide	Methysergide administered: 1) 1–2 mg perorally 30–40 min before DMT; 2) 0.5 mg intramuscularly 10 min before DMT.	*N* = 15 (healthy)	Intensification of DMT subjective effects when taken with methysergide. (1) Two individuals: heightened subjective effects of DMT; five individuals: very intense aggravation of DMT subjective effects. (2) Four individuals who took 65–80% less DMT than their first time experienced similar effects. Four individuals who took 50–60% of their first-time dose did not experience a more pronounced hallucinatory state than their first experience, and in one case, the effects were even less pronounced.
Callaway and Grob (1998)	Case report	Antidepressants (SSRI): fluoxetine	Fluoxetine (20 mg/day) treatment, administration of ~100 ml ayahuasca brew.	*N* = 1 36-year-old male with mild depression	Adverse effects are similar to serotonin toxicity (sweating, shivering, tremors, confusion, severe nausea, vomiting and disorientation) after consumption of ayahuasca brew that lasted for 4 h.

DMT: N,N-dimethyltryptamine; MAOI: monoamine oxidase inhibitor; SSRI: selective serotonin reuptake inhibitor.

**Table 5.** Summary of studies and case reports describing interactions (or the absence thereof) between 5-MeO-DMT and other drugs.

Author and year	Study type	Drug	Dose	Participants	Outcome
Brush et al. (2004)	Case report	Other: β-carbolines	Co-administration of three Syrian rue seeds extract and 10 mg (smoked) + 15–20 mg (snorted) of 5-MeO-DMT.	*N* = 1 17-year-old male	Severe agitation, hallucinations, emesis, rhabdomyolysis, diaphoretic skin, hyperthermia (40.7 °C) and tachycardia (186 bpm).
Sklerov et al. (2005)	Case report	Other: β-carbolines	Unknown composition and quantity of an herbal/synthetic combination, likely containing 5-MeO-DMT (potentially synthetic), DMT and β-carbolines.	*N* = 1 25-year-old male	The subject consumed some type of ‘herbal tonic’ before going to sleep and was found dead the next morning. Autopsy and toxicology examination reported high concentrations of DMT, 5-MeO-DMT, tetrahydroharmine, harmaline and harmine in tissues and biological fluids including liver and blood.

DMT: N,N-dimethyltryptamine; 5-MeO-DMT: 5-methoxy-N,N-dimethyltryptamine.

## Discussion

In this systematic review, a total of 7102 records were screened and 36 reports from 52 studies were included, providing information about the potential DDIs (or the lack of) of classic psychedelics in the scientific literature. Considering the scarcity of studies addressing DDIs involving classic psychedelics, case reports were included to provide more insights into DDIs. Although anecdotal in nature, these case reports highlight potential DDIs and areas for future research. In the following sections, all drugs are discussed, grouped by their properties and/or class, where possible. Groups with blocking or reduced effects on psychedelics are generally addressed first, followed by those with potentiating effects, and concluding with those that have mixed evidence or no effects.

### Serotonin 2A receptor antagonists

Classic psychedelics are known to bind to 5-HT_2A_ receptors where it is thought that their hallucinogenic properties are mediated (Vollenweider and Smallridge, 2022). The results of multiple studies on drugs that are 5-HT_2A_ receptor antagonists, such as ketanserin, trazodone, risperidone and chlorpromazine, indicated that they were all effective in attenuating the effects of LSD and psilocybin. Specifically, ketanserin, a 5-HT_2A_ antagonist with a strong affinity (Becker et al., 2023) was found in all studies to either fully block or reduce the subjective effects of LSD (Becker et al., 2023; Holze et al., 2021; Olbrich et al., 2021; Preller et al., 2018) and psilocybin (Carter et al., 2005, 2007; F. X. Vollenweider et al., 1998). Similarly, decreased physical effects were also observed, including lowered blood pressure, body temperature and heart rate (Holze et al., 2021). Similarly, **risperidone**, a 5-HT_2A_ but also D_2_ antagonist was found to attenuate the effects of psilocybin in a dose-dependent manner (F. X. Vollenweider et al., 1998). **Trazodone**, another 5-HT_2A_ antagonist (Balsara et al., 2005), was shown to reduce the psychological and hallucinogenic effects of LSD in one case report (Bonson et al., 1996).

The findings from studies using chlorpromazine, which is also a 5-HT_2A_ receptor antagonist but has an affinity for D_2_ and adrenoceptor α_1A_ and α_1B_ subtypes as well (Boyd-Kimball et al., 2019; Gillman, 1999), are inconsistent. While two studies (Isbell and Logan, 1957; Murphree, 1962) found that chlorpromazine reduced the intensity of LSD-induced effects as well as anxiety, another two independent studies (Abramson et al., 1960; Schwarz, 1967) reported the opposite. In addition, while Isbell and Logan (1957) reported that oral pre-administration of chlorpromazine blocked the effects of LSD and not post-administration, then Abramson et al. (1960) observed enhanced effects regardless of whether it was administered before or after LSD intake. However, the inconsistent findings regarding the effects of chlorpromazine on LSD response may be due to differences in study design, dosing regimens and individual variability. Moreover, chlorpromazine was reported to attenuate the effects of psilocybin and mescaline, where its pretreatment significantly decreased psilocybin-induced visual perception distortion (Keeler, 1967) and abolished the increased anxiety observed in schizophrenic patients following mescaline ingestion (Lesse, 1958). Overall, considering the available evidence, treatment with potent 5-HT_2A_ antagonists, including medicines, is anticipated to diminish the effects of psychedelics (e.g. antipsychotics risperidone, olanzapine and pipamperone, antidepressants mirtazapine, mianserin and etoperidone as well as migraine prevention drug pizotifen).

### Serotonin 1A receptor agonists

Pretreatment with buspirone, which is a 5-HT_1A_ partial agonist, markedly reduced the psilocybin-induced visual hallucinations (Pokorny et al., 2016). On the contrary, agonism of 5-HT_1A_ with ergotamine did not affect any of the psilocybin effects (Pokorny et al., 2016). However, given that ergotamine has lower efficacy at receptor signal transduction when compared to buspirone and its generally low bioavailability, it was suggested that the administered dose may not have been high enough to compete with psilocybin at those receptor site (Pokorny et al., 2016). These findings indicate that 5-HT_1A_ receptors may be also involved in the manifestation of psilocybin-induced effects such as visual hallucinations, affective alterations, derealisation and depersonalisation. The mechanism of action by which buspirone reduces hallucinations may involve direct stimulation of 5-HT_1A_ receptors, or alternatively, interaction between 5-HT_1A_ and 5-HT_2A_ receptors on pyramidal cells due to co-expression of 5-HT_1A_ and 5-HT_2A_ receptors in cortical and visual areas (Pokorny et al., 2016). Despite both psilocin and buspirone being partial agonists at 5-HT_1A_ receptors, the blocking effect of buspirone may be due to a more effective inhibitory impact on pyramidal neurons compared to psilocin (Pokorny et al., 2016).

### Dopamine receptor antagonists

Buspirone is also an antagonist for dopamine D_2_ receptors (low affinity) and has shown weak affinity to the 5-HT_2_ receptors (Loane and Politis, 2012), which could have a role in modulating the effects of psilocybin as well. However, while LSD is known to have an effect on the dopaminergic system (found to be important in perceived selfhood and cognition) (Lawn et al., 2022) and it has a high affinity for both D_1_ and D_2_ receptors (Halberstadt and Geyer, 2011), then psilocin does not bind to dopamine receptors (Halberstadt and Geyer, 2011). Moreover, D_2_ antagonism by haloperidol alone did not have an effect on psilocybin-induced hallucinations (F. X. Vollenweider et al., 1998). Haloperidol did, however, diminish the feelings of oceanic boundlessness and derealisation and also increased anxiety (F. X. Vollenweider et al., 1998). In this case, psilocybin may exert an indirect impact on dopaminergic systems, which is subsequently counteracted by haloperidol (F. X. Vollenweider et al., 1998). It has been shown in rats that psilocybin administration can increase extracellular dopamine levels in the frontal cortex (Wojtas et al., 2022) and psilocin administration has also increased dopamine levels in the nucleus accumbens (Sakashita et al., 2015).

### Serotonin reuptake inhibitors

Interestingly, the blocking of serotonin reuptake transporters with SSRIs, such as fluoxetine, sertraline and paroxetine, reduced the effects of LSD as reported in one study (Bonson et al., 1996). Moreover, fluoxetine also delayed the onset of LSD effects in nearly half of the participants in the same study (Bonson et al., 1996) and was shown to markedly decrease sensitivity to LSD in one case report (Strassman, 1992). SSRIs can also result in DDI at the pharmacokinetic level as some of them, such as fluoxetine, are potent inhibitors of CYP2D6 enzymes (Brøsen, 1998), which can modulate the effects of psychedelics that are being metabolised by such enzymes. For example, CYP2D6 enzymatic activity is already known to influence the effects of LSD (Straumann et al., 2023; Vizeli et al., 2021). Considering this, one might have expected a stronger and more prolonged response from LSD instead of reduced effects. However, ultimately this could involve an interplay between various molecular actions, including interactions with enzymes, receptor availability and other molecular factors.

Trazodone, which was previously described to reduce LSD effects as it is a 5-HT_2A_ antagonist, is also a serotonin receptor antagonist and reuptake inhibitor (SARI) (Fagiolini et al., 2012) that might have a role in the observed effects as well. Potentially, SSRIs and SARIs that increase extracellular serotonin by inhibiting its uptake can then attenuate the effects of psychedelics via receptor competition with the endogenous serotonin. In addition, it has been shown that repeated administration of SSRIs desensitises 5-HT_2A_ receptors which may reduce the cell’s response to psychedelics by binding to these receptors (Gray and Roth, 2001). Chronic SSRI use can also increase serotonin release via desensitisation of 5-HT_1A_ raphe autoreceptors (Artigas et al., 2001).

However, one SSRI, escitalopram, did not have an effect on psilocybin-induced positive mood or mind-altering effects (depersonalisation, oceanic boundlessness or euphoria), but it did reduce ego disintegration and anxiety (Becker et al., 2022). The authors hypothesised that while LSD does not affect the serotonin transporter, psilocin has a weak inhibitory effect on it (Becker et al., 2022; Rickli et al., 2016). This distinction in pharmacology could lead to different interactions between antidepressants and LSD compared to psilocybin (Becker et al., 2022), explaining the differences in response. Although this study offers important insights into the safety of administering psilocybin to patients taking escitalopram, it is crucial to bear in mind that the treatment phase of this study was 14 days. Longer treatment periods with escitalopram, which may often last for years, could lead to distinct outcomes due to molecular changes that occur over extended periods (Faure et al., 2006).

Nonetheless, another study (not included in the results) involved 19 patients with treatment-resistant depression and who were on chronic SSRI treatment (sertraline, escitalopram, fluoxetine, vilazodone, paroxetine or citalopram). The patients were treated with synthetic psilocybin (an investigational drug named ‘COMP360’) and no serious treatment-emergent adverse events were reported (Goodwin et al., 2023). However, two cases were considered severe (both blood pressure increase) and necessitated treatment with clonidine, but the study did not provide information regarding which specific SSRI treatments these individuals were on. Secondly, although the study measured 5-dimensional altered states of consciousness (5D-ASC) scores, it also did not provide a breakdown by specific SSRIs. This absence of information makes it difficult to assess and compare the individual impacts of different SSRIs on subjective effects as well, particularly given the high standard deviations in the 5D-ASC results and instances of zero scores in three dimensions, indicating some participants did not respond to psilocybin. Improvements in depression severity were observed in 42.1% of cases at week 3, which indicates that ongoing SSRI treatment did not significantly hinder psilocybin’s therapeutic potential, at least for those participants. Based on the results, the authors hypothesise that chronic SSRI treatment may not significantly downregulate 5-HT_2A/C_ receptors, or potentially indicate a lesser impact on the downregulated receptors on the psychedelic experience than previously theorised. It is also possible that unknown mechanisms downstream of 5-HT_2A/C_ receptor signalling could compensate for any potential effects of chronic SSRI treatment on the psychedelic response, as they state (Goodwin et al., 2023). While the study contributes to the body of evidence supporting the safety of using psilocybin alongside SSRI treatment, it is important to note its limitations, including a small sample size and the absence of a breakdown of specific SSRIs. This latter is crucial for linking adverse events and subjective effects to individual drugs, given their distinct properties and potential for varied drug interactions. Due to this, this article was not included in the main results.

Another study by Gukasyan et al. (2023), based on online retrospective survey results, also provided generalised findings for SSRIs and serotonin and norepinephrine reuptake inhibitors (SNRIs) (thus not included in the primary results). This study reported weakened effects of psilocybin in approximately half of the participants when used concurrently with SSRI/SNRIs, also suggesting downregulation of 5-HT_2A_ receptors (Gukasyan et al., 2023).

### Monoamine oxidase inhibitors

Results from another antidepressant class, MAOIs, showed a similar outcome to those of SSRIs. In particular, phenelzine, isocarboxazid, nialamide and iproniazid are all non-selective MAO inhibitors (Entzeroth and Ratty, 2017), therefore inhibiting both MAO-A and MAO-B enzymes. The first three listed antidepressants attenuated or blocked the effects of LSD (Bonson and Murphy, 1996; Grof and Dytrych, 1965; Resnick et al., 1964). By contrast, the latter, iproniazid, did not alter the subjective or physical effects of LSD (DeMaar et al., 1960). However, iproniazid was also used in the experiments with DMT where it was shown to reduce the effects of DMT (Sai Halasz, 1963). This particular finding is interesting as iproniazid is an irreversible MAOI (Entzeroth and Ratty, 2017) that inhibits the enzyme that rapidly metabolises DMT (Barker et al., 1980). Moreover, studies in rats have shown that iproniazid pretreatment increases levels of DMT in the brain, as well as in the liver, kidney and blood (Sitaram et al., 1987). However, the reduced DMT effects could be due to increased serotonin levels after blocking MAO and it is hypothesised that higher doses of DMT are needed when serotonin levels are elevated (Sai Halasz, 1963). It is suggested that ayahuasca’s effect is mediated by MAO inhibition in the digestive system or bloodstream, which protects DMT from metabolism during its transit to the brain, where MAO inhibitors can then attenuate DMT’s effects due to elevated brain serotonin (Ott, 1996). This hypothesis for DMT can also explain the similar, attenuated response, which was observed in the studies where LSD was combined with MAOIs (Bonson and Murphy, 1996; Grof and Dytrych, 1965; Resnick et al., 1964).

In addition to metabolising DMT, MAOIs are also important in the breakdown of serotonin as they block MAO enzymes involved in its metabolism (Foong et al., 2018). This gives rise to a possible scenario of excessive serotonin levels in the brain. In particular, when combining MAOIs with each other or with SSRI/SNRI, it has been thought to carry the greatest risk of serotonin toxicity (Foong et al., 2018). In one case report, symptoms resembling serotonin toxicity were reported after an individual was administered ayahuasca while being on a fluoxetine treatment (Callaway and Grob, 1998). However, the patient also recovered rapidly within 4 h after the administration of ayahuasca without any treatment. Moreover, another case report that was not included in the review documented an instance of serotonin toxicity in an individual who had been undergoing **fluoxetine** and **quetiapine** treatment while consuming pure harmal extracted from *Peganum harmala*, which is a component of ayahuasca (Bakim et al., 2012).

Ayahuasca brew is usually made from a plant containing DMT and another plant that contains β-carbolines which mostly inhibit MAO-A, thereby making DMT orally active (McKenna, 2004). Tetrahydroharmine (THH), one of the β-carbolines present in the brew, can also act as a weak serotonin uptake inhibitor and increase brain serotonin levels (Airaksinen et al., 1980). Fluoxetine strongly inhibits the CYP2D6 enzyme, which metabolises many drugs, including serotonin. The concurrent inhibition of serotonin reuptake and serotonin-metabolising enzymes, such as CYP2D6 and MAO, can cause an accumulation of serotonin in the brain and low clearance, potentially leading to life-threatening serotonin toxicity (Dunkley et al., 2003). This explanation aligns with the reported case reports; however, further research is necessary to confirm the potential association. While more evidence is required to shed light on the possible DDI, it would be advisable to exercise caution when combining MAOIs found in ayahuasca with SSRIs and SNRIs (e.g. citalopram, escitalopram, fluvoxamine, fluoxetine, paroxetine and sertraline).

There are two case reports in which the consumption of 5-MeO-DMT alongside β-carbolines (MAO-A inhibitors) resulted in a serious adverse event. In one case (Brush et al., 2004), the ingestion of 5-MeO-DMT in combination with three Syrian rue (*Peganum harmala*) seeds containing harmaline and harmine led to severe agitation, hallucinations, emesis, rhabdomyolysis, diaphoretic skin, hyperthermia and tachycardia. Based on the observed time course and symptoms, the authors suggested the possibility of MAOI poisoning (Brush et al., 2004). Alternatively, they also considered serotonin toxicity but deemed it less likely. A critical assessment of the report by dos Santos (2013) suggested serotonin toxicity over MAOI poisoning (dos Santos, 2013).

In this case report, it was indicated that three Syrian rue seeds were consumed. However, considering the tiny weight (Li et al., 2023) and the average content of alkaloids in these seeds (Herraiz et al., 2010), the estimated total harmaline and harmine content would be only approximately 0.26 mg and 0.34 mg, respectively. The combined amount is at least 50 times less than the same alkaloid levels typically used in a standard ayahuasca brew for MAO inhibition (D. J. McKenna et al., 1984; Rivier and Lindgren, 1972). While it is challenging to determine the minimum dose of harmaline/harmine necessary for MAO inhibition that induces such adverse effects, it is important to approach this reported consumption of three seeds with a degree of scepticism. It might be possible that the individual may have instead consumed seed capsules, each containing over 50 seeds (Moloudizargari et al., 2013), totalling approximately over 30 mg of combined harmaline and harmine for three capsules, comparable to doses used in an ayahuasca brew.

The second case report (Sklerov et al., 2005) described the death of a 25-year-old male after he consumed herbal extracts containing β-carbolines and tryptamines. No information is available regarding the composition and dosage of the products but toxicology analysis revealed the presence of 5-MeO-DMT, DMT, MAOIs (harmaline and harmine) and THH (weak serotonin uptake inhibitor). This particular report has faced some criticism for associating toxicity with ayahuasca and for not including several important details (Callaway et al., 2006). Callaway and his colleagues suggested that the elevated levels of 5-MeO-DMT found in the deceased individual’s blood likely have a synthetic origin rather than originating from an ayahuasca brew. This conclusion arises because common ingredients in ayahuasca brew do not contain 5-MeO-DMT or, at most, contain only trace amounts of it (Callaway et al., 2006).

The toxicity observed in those two case reports remains unclear, but could also have arisen from the impact of MAOIs on the levels of 5-MeO-DMT in the blood and brain as hypothesised by Halberstadt (2016). Inhibiting MAO-A activity by β-carbolines could substantially increase concentrations and accumulation of 5-MeO-DMT in the brain and may also lead to interactions with various other targets, potentially giving rise to adverse effects and toxicity (Halberstadt, 2016; Jiang et al., 2013). Further evidence is required to confirm the link between 5-MeO-DMT and MAO-A inhibitors. However, based on the case reports, one should exercise caution when combining 5-MeO-DMT with either MAO-A inhibitors (such as harmaline and harmine) or non-selective MAO inhibitors (e.g. medicines such as tranylcypromine, isocarboxazid, phenelzine and selegiline).

### Other drugs (blocking action)

One study that reported a blocking action on LSD effects was using a high dose of niacin (vitamin B3). Pretreatment with niacin resulted in delayed onset of LSD effects and prevented most of the perceptual changes from occurring, and administration post-LSD ingestion attenuated all effects of LSD within 5 min (Agnew and Hoffer, 1955). The blocking mechanism of niacin is not clear but may be due to increased serotonin levels. This is indicated by the studies where niacin has been shown to induce serotonin release from human platelets within a few minutes and to increase plasma serotonin levels in rats (Papaliodis et al., 2008). Secondly, blocking 5-HT_2A_ receptors with ketanserin inhibits niacin-induced temperature increase (Papaliodis et al., 2008), which indicates that serotonin is involved in the effects of niacin. Finally, high-dose treatment with niacin has caused manic-like psychotic episodes that were thought to occur via stimulation of serotonin but also dopamine production (Loebl and Raskin, 2013).

### Tricyclic antidepressants

Several studies have reported the potentiated effects of LSD. In one study, desipramine, imipramine and clomipramine, which are all TCAs, were shown to increase the psychological effects of LSD (Bonson and Murphy, 1996). Desipramine is a potent inhibitor of noradrenaline reuptake, while imipramine and clomipramine exhibit a lower degree of noradrenaline and serotonin reuptake inhibition (Gillman, 2007). Furthermore, clomipramine also functions as a 5-HT_2A_ receptor antagonist and also imipramine has an affinity for this receptor subtype (Gillman, 2007).

Since TCAs were reported to enhance the effects of LSD despite some of them inhibiting serotonin uptake and are even 5-HT_2A_ receptor antagonists (similar to SSRIs that decreased the effects of LSD), it is suggested another mechanism can contribute. For instance, chronic administration of TCAs, such as desipramine, can increase the sensitivity of certain neurons to LSD, suggesting that these medications may sensitise postsynaptic serotonin receptors in the brain and therefore be more responsive to LSD (Bonson and Murphy, 1996). Alternatively, the effects can be enhanced through a dopaminergic system (Bonson and Murphy, 1996) as chronic use of desipramine has been demonstrated to result in an elevated behavioural response to amphetamine, whereas chronic use of fluoxetine did not have the same effect (Spyraki and Fibiger, 1981). Therefore, it is possible that the observed effects are due to a modification in the sensitivity of the dopamine receptors. However, it is noteworthy that the results of TCAs originate from a single study with a very small number of participants.

### Other drugs (potentiating action)

Additionally, from the same study, it was reported that chronic lithium use potentiated LSD effects and resulted in earlier onset (Bonson and Murphy, 1996). It has been suggested that while acute administration of lithium increases serotonin levels in the brain, chronic administration on the other hand reduces serotonin concentrations (Bonson and Murphy, 1996). This may explain why long-term lithium use enhances LSD effects, as LSD acts as an agonist in the absence of endogenous serotonin resulting in the observed behavioural effects (Bonson and Murphy, 1996). It is important to note that these findings and some anecdotal reports that can be found on internet forums suggest a similar outcome, there are also several reported instances of lithium causing seizures when used concurrently with LSD or psilocybin (Nayak et al., 2021).

Intensified and prolonged (negative) effects of LSD have also been reported in individuals being pretreated with reserpine, an antihypertensive drug, in three separate studies (Freedman, 1963; Isbell and Logan, 1957; Resnick et al., 1965). Both Freedman (1963) and Isbell and Logan (1957) reported tremors that were present after using LSD together with reserpine. Tremors were not observed during LSD use alone (Isbell and Logan, 1957), and when combined with reserpine, the experience was reported to be less pleasant and lasted longer than LSD use alone (Freedman, 1963). Furthermore, specific types of hallucinations were reported with reserpine treatment that were not observed during LSD use alone. Overall, individuals reported unpleasant experiences when using an LSD with reserpine.

Reserpine has the ability to bind to the storage vesicles of certain neurotransmitters, including dopamine and norepinephrine. This binding inhibits the catecholamine pumps and blocks the uptake of serotonin, norepinephrine and dopamine into the presynaptic storage vesicles. Ultimately, this leads to the depletion of these neurotransmitters by cytoplasmic MAO at both central and peripheral synapses (Cheung and Parmar, 2023). Lower serotonin levels can enhance LSD effects, which was also observed for lithium. However, when LSD was used after chronic administration of lithium, it resulted in positive effects and more vivid hallucinations, without any noted increase in negative psychological or physical side effects as for reserpine. Treatment with reserpine has been known to cause several neurological side effects (Cheung and Parmar, 2023), and studies have shown that in patients with anxiety it can exacerbate their symptoms (Peterfy et al., 1976; Sarwer-Foner and Ogle, 1956). Since LSD can also increase anxiety as a side effect (Strassman, 1984), it is possible that reserpine treatment could enhance this effect (or *vice versa*), while the modulated hallucinogenic effects of LSD are due to depleted serotonin levels.

Moreover, reserpine is also a potent inhibitor of both CYP2C19 and CYP2D6 enzymes (Englund et al., 2014), which are involved in the initial metabolic steps of LSD (Wagmann et al., 2019). This inhibition may account for the reported prolonged effects of LSD when it was used with reserpine (Freedman, 1963) as it could potentially decrease the metabolism of LSD. Finally, reserpine acts as an inhibitor of P-glycoprotein as well (Englund et al., 2014), which can lead to increased concentrations of P-gp substrates. However, no research has been conducted to investigate the impact of P-gp activity on LSD pharmacokinetics and its effects, making it difficult to assess the potential contribution of P-gp inhibition.

Racemic pindolol, which has an affinity for 5-HT_1A_ and β-adrenergic receptors (Artigas et al., 2001), was reported to intensify the effects of DMT (Strassman, 1996). The authors suggested a buffering effect of 5-HT_1A_ that blocked the 5-HT_2_-mediated psychedelic effects (Strassman, 1996). However, pindolol has been shown to accelerate, and in some cases, enhance the antidepressant effects of SSRIs. This can be mediated by antagonising 5-HT_1A_ autoreceptors in the midbrain raphe and, as a result, preventing the inhibition of serotonin release (Artigas et al., 2001). DMT is a full agonist at 5-HT_1A_ receptors and has a higher affinity for the receptor than it has for 5-HT_2A_ receptors where it is a weak partial agonist (Kozell et al., 2023). Hypothetically, a similar effect as for SSRIs has been described, and can also modulate the effects of DMT, which may have enhanced effects via activating postsynaptic 5-HT_1A_ receptors while 5-HT_1A_ autoreceptors are blocked.

A 5-HT_1_ receptor agonist and 5-HT_2_ receptor antagonist methysergide were similarly shown to potentiate DMT’s effects (Sai-Halasz, 1962). This finding adds more weight to the 5-HT_1A_ receptor contribution to the effects of DMT, especially considering that methysergide is also an antagonist at the 5-HT_2A_ receptors.

With regards to mescaline use and potentiated effects, one case report involved the use of 2C-O, a structural isomer of mescaline that did not appear to have psychedelic properties on its own (Dittrich, 1971). However, when used together with mescaline, a synergistic effect may have occurred that potentiated the effects of mescaline described in the case report.

### Other drugs (mixed results)

Azacyclonol, once investigated as a potential antidepressant, was experimented on individuals who administered LSD and mescaline. Two studies showed no effect of blocking LSD’s actions (Clark, 1956; Isbell and Logan, 1957) and one case report describes the same for mescaline (Clark, 1956). These results are in contrast to another study that reported the attenuated (pretreatment) or blocked (acute) effects of mescaline after azacyclonol treatment (Fabing, 1955). The mechanism of action of azacyclonol is not well understood, and the mixed results from the described studies make it difficult to draw firm conclusions and hypotheses.

### Recreational drugs

Finally, two studies reported the effects of recreational drugs, including MDMA and alcohol consumption on LSD and alcohol on psilocybin. One study, which investigated the effects of alcohol, found that LSD and psilocybin (to a lesser extent) acted as antagonists to the subjective effects of alcohol, while their own psychedelic effects were mainly unaffected (Barrett et al., 2000). It has been suggested that LSD may block the subjective effects of alcohol by interacting with 5-HT_1B_ and 5-HT_1C_ receptors, which are implicated in the formation of the ethanol cue (Barrett et al., 2000). Evidence shows that agonists of these receptors produce responses similar to those of alcohol, whereas blockade of these receptors interferes with the discriminative stimulus properties of alcohol (Barrett et al., 2000). In the second, a placebo-controlled study (Straumann et al., 2023), LSD was co-administered with MDMA to investigate the benefits of a combination also known as ‘candy-flipping’. While the particular combination did not yield significant changes as assessed by various instruments, it did result in longer-lasting drug effects (averaging 1.5 h longer, compared to LSD alone). Also, the combination led to higher plasma concentrations and an extended plasma elimination half-life for LSD, compared to LSD alone. The prolonged drug effects could be attributed to decreased CYP2D6 activity, an enzyme known to be inhibited by MDMA (O’Mathúna et al., 2008) and involved in LSD metabolism (Vizeli et al., 2021).

### Limitations

One of the limitations of this study is the inclusion of a number of old research articles, particularly those published between the 1950s and the 1970s, where many of them provided limited information about the outcomes and/or methods used. Additionally, the limited number of total studies included in this review led to the inclusion of case reports, which may be subject to bias and may provide limited generalisability to larger populations. This review may also have also missed some relevant studies that were published only in non-English languages, which were more common in the early days of research. Finally, this review focused on interactions with LSD, psilocybin, mescaline, 5-MeO-DMT, DMT and ayahuasca, while not including other psychedelics.

## Conclusions

In this systematic review, we observed DDIs at both pharmacodynamic and (likely) pharmacokinetic levels that may block or decrease the response to psychedelics, or alternatively potentiate and lengthen the duration of psychological and/or physical effects. While there is strong evidence of 5-HT_2A_ receptor involvement in the effects of psychedelics, some research included in this review suggests that other serotonin receptors, such as 5-HT_1A/B_ and dopamine receptors, along with altered serotonin levels, may also modulate psychological and/or physical effects. Additionally, a small number of studies reviewed indicated a potential role of the 5-HT_1_ receptor subtype in modulating the effects of DMT. It appears that although different psychedelics may yield similar subjective effects, their pharmacological properties differ, resulting in potentially varying interaction effects when combined with other drugs. Overall, given the limited number of papers exploring DDIs associated with psychedelics and the resurgence of scientific and medical interest in these compounds, further research is needed to improve understanding of such interactions, and identify novel drug interactions and potentially serious adverse reactions not currently described in the literature.

## Supplemental Material

sj-docx-1-jop-10.1177_02698811231211219 – Supplemental material for Drug–drug interactions involving classic psychedelics: A systematic review Click here for additional data file. Supplemental material, sj-docx-1-jop-10.1177_02698811231211219 for Drug–drug interactions involving classic psychedelics: A systematic review by Andreas Halman, Geraldine Kong, Jerome Sarris and Daniel Perkins in Journal of Psychopharmacology
