# Supplementary material for: Drug–drug interactions involving classic psychedelics: A systematic review
Source: J Psychopharmacol. 2023 Nov 20;38(1):3–18. doi: 10.1177/02698811231211219 (PMC10851641; doi:10.1177/02698811231211219)
Supplement: sj-docx-1-jop-10.1177_02698811231211219 – Supplemental material for Drug–drug interactions involving classic psychedelics: A systematic review [file sj-docx-1-jop-10.1177_02698811231211219.docx]

Supplementary Information

### Text S1. Search terms for LSD, psilocybin, mescaline, DMT and ayahuasca.

The following search was used in PubMed:

( "Lysergic Acid Diethylamide"[MeSH] OR "lysergic acid diethylamide*"[Title/Abstract] OR "LSD"[Title/Abstract] OR "L.S.D."[Title/Abstract] OR "Psilocybin"[MeSH] OR "psilocybin"[Title/Abstract] OR "psilocin"[Title/Abstract] OR "magic mushroom*"[Title/Abstract] OR "Psilocybe"[Title/Abstract] OR "Mescaline"[MeSH] OR "mescalin*"[Title/Abstract] OR "peyote"[Title/Abstract] OR "san pedro"[Title/Abstract] OR "3,4,5-trimethoxyphenethylamine"[Title/Abstract] OR "trimethoxyphenethylamine"[Title/Abstract] OR "N,N-Dimethyltryptamine"[MeSH] OR "dimethyltryptamine"[Title/Abstract] OR "DMT"[Title/Abstract] OR "D.M.T."[Title/Abstract] OR "ayahuasca"[Title/Abstract] OR "harmaline"[Title/Abstract] OR "harmine"[Title/Abstract] OR "banisteriopsis"[Title/Abstract] OR "psychotria"[Title/Abstract] OR "chacruna"[Title/Abstract] OR "diplopterys"[Title/Abstract] OR "chaliponga"[Title/Abstract] OR "chagropanga"[Title/Abstract] OR "syrian rue"[Title/Abstract] OR "peganum"[Title/Abstract] OR "mimosa"[Title/Abstract] OR "jurema"[Title/Abstract] OR "acacia"[Title/Abstract] ) AND ( "Drug Interactions"[MeSH] OR "Drug-Related Side Effects and Adverse Reactions"[MeSH] OR "Pharmacokinetics"[MeSH] OR "drug-drug"[Text Word] OR "co-administrat*"[Text Word] OR "co-ingest*"[Text Word] OR "interaction"[Text Word] OR "pharmacodynamic*"[Text Word] OR "pharmacokinetic*"[Text Word] ) NOT ( "rat"[Title] OR "rats"[Title] OR "mouse"[Title] OR "mice"[Title] OR "murine"[Title] OR "cat"[Title] OR "cats"[Title] OR "dog"[Title] OR "dogs"[Title] ) AND ( "journal article"[Publication Type] OR "randomized controlled trial"[Publication Type] OR "clinical study"[Publication Type] OR "clinical trial"[Publication Type] OR "clinical trial, phase i"[Publication Type] OR "clinical trial, phase ii"[Publication Type] OR "clinical trial, phase iii"[Publication Type] OR "clinical trial, phase iv"[Publication Type] OR "controlled clinical trial"[Publication Type] OR "pragmatic clinical trial"[Publication Type] OR "comparative study"[Publication Type] OR "observational study"[Publication Type] OR "case reports"[Publication Type] ) NOT ( "review"[Publication Type] OR "preprint"[Publication Type] ) AND ( "english"[Language] )

The following search was used in Web of Science:

TS=("lysergic acid diethylamide*" OR LSD OR "L.S.D." OR psilocybin OR psilocin OR "magic mushroom*" OR Psilocybe OR mescalin* OR peyote OR "san pedro" OR "3,4,5-trimethoxyphenethylamine" OR "trimethoxyphenethylamine" OR "N,N-Dimethyltryptamine" OR "dimethyltryptamine" OR "DMT" OR "D.M.T" OR harmaline OR harmine OR ayahuasca OR banisteriopsis OR psychotria OR chacruna OR diplopterys OR chaliponga OR chagropanga OR "syrian rue" OR peganum OR mimosa OR jurema OR acacia) AND ALL=(drug-drug OR co-administrat* OR co-ingest* OR interaction OR pharmacodynamic* OR pharmacokinetic*) NOT TI=(rat OR rats OR mouse OR mice OR murine OR cat OR cats OR dog OR dogs) AND DT=(Article) AND LA=(English) AND SU=(Pharmacology OR Pharmacy OR Neurosciences OR Psychiatry OR Clinical Neurology OR Biochemistry Molecular Biology OR Chemistry Medicinal OR Psychology Clinical OR Toxicology OR Multidisciplinary Sciences OR Psychology OR Substance Abuse OR Medicine Research Experimental OR Behavioral Sciences OR Biology OR Integrative Complementary Medicine OR Cell Biology OR Medicine General Internal OR Psychology Biological OR Public Environmental Occupational Health OR Medicine Legal OR Physiology OR Immunology OR Psychology Experimental OR Pediatrics OR Emergency Medicine OR Medical Ethics OR Genetics Heredity)

The following search was used in PsycINFO:

(

exp "lysergic acid diethylamide"/ or

"lysergic acid diethylamide*".ti,ab. or

"LSD".ti,ab. or

"L.S.D.".ti,ab. or

exp "psilocybin"/ or

"psilocybin".ti,ab. or

"psilocin".ti,ab. or

"magic mushroom*".ti,ab. or

"psilocybe".ti,ab. or

exp "mescaline"/ or

"mescalin*".ti,ab. or

"peyote".ti,ab. or

"san pedro".ti,ab. or

"3,4,5-trimethoxyphenethylamine".ti,ab. or

"trimethoxyphenethylamine".ti,ab. or

exp "n,n-dimethyltryptamine"/ or

"dimethyltryptamine".ti,ab. or

"dmt".ti,ab. or

"d.m.t.".ti,ab. or

"ayahuasca".ti,ab. or

"harmaline".ti,ab. or

"harmine".ti,ab. or

"banisteriopsis".ti,ab. or

"psychotria".ti,ab. or

"chacruna".ti,ab. or

"diplopterys".ti,ab. or

"chaliponga".ti,ab. or

"chagropanga".ti,ab. or

"syrian rue".ti,ab. or

"peganum".ti,ab. or

"mimosa".ti,ab. or

"jurema".ti,ab. or

"acacia".ti,ab.

)

and

(

exp "drug interactions"/ or

exp "drug-related side effects and exp adverse reactions"/ or

exp "pharmacokinetics"/ or

"drug-drug".tw. or

"co-administrat*".tw. or

"co-ingest*".tw. or

"interaction".tw. or

"pharmacodynamic*".tw. or

"pharmacokinetic*".tw.

)

not

(

"rat".ti or

"rats".ti or

"mouse".ti or

"mice".ti or

"murine".ti or

"cat".ti or

"cats".ti or

"dog".ti or

"dogs".ti

)

*Search results limited to: Journal article or Reprint (Document type); Humans (Population group); English (Language)*

### Text S2. Search terms for 5-MeO-DMT.

The following search was used in PubMed:

( "Methoxydimethyltryptamines"[MeSH] OR "5-methoxy-N,N-dimethyltryptamine"[Title/Abstract] OR "5-MeO-DMT"[Title/Abstract] ) NOT ( "rat"[Title] OR "rats"[Title] OR "mouse"[Title] OR "mice"[Title] OR "murine"[Title] OR "cat"[Title] OR "cats"[Title] OR "dog"[Title] OR "dogs"[Title] ) AND ( "journal article"[Publication Type] OR "randomized controlled trial"[Publication Type] OR "clinical study"[Publication Type] OR "clinical trial"[Publication Type] OR "clinical trial, phase i"[Publication Type] OR "clinical trial, phase ii"[Publication Type] OR "clinical trial, phase iii"[Publication Type] OR "clinical trial, phase iv"[Publication Type] OR "controlled clinical trial"[Publication Type] OR "pragmatic clinical trial"[Publication Type] OR "comparative study"[Publication Type] OR "observational study"[Publication Type] OR "case reports"[Publication Type] ) NOT ( "review"[Publication Type] OR "preprint"[Publication Type] ) AND ( "english"[Language] )

The following search was used in Web of Science:

TS=("5-MeO-DMT" OR "5-methoxy-N,N-dimethyltryptamine") NOT TI=(rat OR rats OR mouse OR mice OR murine OR cat OR cats OR dog OR dogs) AND DT=(Article) AND LA=(English) AND SU=(Pharmacology OR Pharmacy OR Neurosciences OR Psychiatry OR Clinical Neurology OR Biochemistry Molecular Biology OR Chemistry Medicinal OR Psychology Clinical OR Toxicology OR Multidisciplinary Sciences OR Psychology OR Substance Abuse OR Medicine Research Experimental OR Behavioral Sciences OR Biology OR Integrative Complementary Medicine OR Cell Biology OR Medicine General Internal OR Psychology Biological OR Public Environmental Occupational Health OR Medicine Legal OR Physiology OR Immunology OR Psychology Experimental OR Pediatrics OR Emergency Medicine OR Medical Ethics OR Genetics Heredity)

The following search was used in PsycINFO:

(

exp "5-methoxy-n,n-dimethyltryptamine"/ or

"5-methoxy-n,n-dimethyltryptamine".ti,ab. or

"5-meo-dmt".ti,ab.

)

not

(

"rat".ti or

"rats".ti or

"mouse".ti or

"mice".ti or

"murine".ti or

"cat".ti or

"cats".ti or

"dog".ti or

"dogs".ti

)

### Table S1. Summary table of all drug-drug interactions together with molecular targets.

| **Class and Drug** | **Drug's targets/actions** | **LSD** | **Psilocybin** | **Mescaline** | **5-MeO-DMT, DMT or ayahuasca** |
| --- | --- | --- | --- | --- | --- |
| **ANXIOLYTICS**  Buspirone | 5-HT_1A_ partial agonist (Pokorny et al., 2016); D_2_ antagonist (low affinity) and weak affinity to 5-HT_2_ (Loane & Politis, 2012) |  | ↓ Pretreatment markedly reduced visual perception distortion, depersonalization and derealisation (Pokorny et al., 2016) |  |  |
| **ANTIPSYCHOTICS**  Chlorpromazine | 5-HT_2A_ antagonist, D_2_ antagonist (Boyd-Kimball et al., 2019) | ↓ Pretreatment reduced intensity and acute blocked LSD effects (H. Isbell & Logan, 1957).  ↓ Pretreatment blocked subjective LSD effects (Murphree, 1962).  ↑ Pre- and post-treatment increased positive LSD effects and reduced anxiety (Abramson et al., 1960).  – Simultaneous ingestions resulted in no differences (Abramson et al., 1960).  ↑ Post-treatment intensified LSD effects and increased anxiety (Schwarz, 1967) | ↓ Pretreatment significantly decreased pupil dilation, visual perception distortion, subjective effects (Keeler, 1967) | ↓ Chlorpromazine and promazine abolished the mounting anxiety in 68% participants (Lesse, 1958) |  |
| Haloperidol | D_2_ antagonist (Vollenweider et al., 1998) |  | ⇅ Diminished the feelings of oceanic boundlessness and derealization; no effect on hallucinations; increased anxiety (Vollenweider et al., 1998) |  |  |
| Risperidone | 5-HT_2A_ antagonist, D_2_ antagonist (Vollenweider et al., 1998) |  | ↓ Attenuated the visual hallucination, depersonalisation and derealisation effects of psilocybin in a dose-dependent manner (Vollenweider et al., 1998) |  |  |
| **MOOD STABILISERS**  Lithium | 5-HT_1B_ binding ability (Massot et al., 1999) | ↑ Chronic lithium use potentiated subjective LSD effects + earlier onset (Bonson & Murphy, 1996) |  |  |  |
| **ANTIDEPRESSANTS**  SSRI:  Fluoxetine | Potent CYP2D6 inhibitor (Sproule et al., 1997),  5-HT_2B_ agonist (Peng et al., 2014) | ↓ Pretreatment delayed onset of LSD effects (44% patients) and markedly diminished the subjective response of LSD (Bonson et al., 1996).  ↓ Markedly decreased in sensitivity to LSD and reduced subjective effects (Strassman, 1992)* |  |  | ✕ Adverse effects similar to serotonin toxicity after consumption of ayahuasca (Callaway & Grob, 1998)* |
| SSRI: Escitalopram | CYP2D6 inhibitor (weak) (Noehr-Jensen et al., 2009)  5-HT_2B_ agonist (Peng et al., 2014) |  | ⇅ Decreased anxiety, but did not reduce the positive mood or mind-altering effects (Becker et al., 2022) |  |  |
| SSRI: Sertraline | CYP2D6 inhibitor (Sproule et al., 1997),  5-HT_2B_ agonist (Peng et al., 2014) | ↓ Pretreatment decreased subjective effects of LSD (Bonson et al., 1996) |  |  |  |
| SSRI: Paroxetine | CYP2D6 inhibitor (potent) (Sproule et al., 1997),  5-HT_2B_ agonist (Peng et al., 2014) | ↓ Pretreatment reduced subjective effects of LSD (Bonson et al., 1996) |  |  |  |
| SARI: Trazodone | 5-HT_2A_ and 5-HT_2C_ antagonist, α_1_- and α_2_-adrenergic receptor antagonist and histamine H_1_ receptor antagonist, SERT inhibitor (Fagiolini et al., 2012) | ↓ Pretreatment reduced subjective effects of LSD (Bonson et al., 1996)* |  |  |  |
| TCA: Desipramine | 5-HT and NA reuptake (high affinity) inhibitor (Gillman, 2007) | ↑ Earlier onset of LSD effects and increased psychological effects (Bonson & Murphy, 1996) |  |  |  |
| TCA: Imipramine | 5-HT and NA reuptake inhibitor, potent CYP2C19 inhibitor (Gillman, 2007) | ↑ Earlier onset of LSD effects and increased psychological effects (Bonson & Murphy, 1996) |  |  |  |
| TCA: Clomipramine | 5-HT (high affinity) and NA reuptake inhibitor, potent CYP2C19 and CYP1A2 inhibitor (Gillman, 2007) | ↑ Earlier onset of LSD effects and increased psychological effects (Bonson & Murphy, 1996)* |  |  |  |
| MAOI: Phenelzine | Irreversible non-selective MAO inhibitor (Entzeroth & Ratty, 2017) | ↓ Nearly abolished the subjective responses of LSD (Bonson & Murphy, 1996) |  |  |  |
| MAOI: Isocarboxazid | Irreversible non-selective MAO inhibitor (Entzeroth & Ratty, 2017) | ↓ Pretreatment attenuated psychological, autonomic and neurologic responses of LSD (Resnick et al., 1964) |  |  |  |
| MAOI: Nialamide | Irreversible non-selective MAO inhibitor (Entzeroth & Ratty, 2017) | ↓ Premedication blocked reactions to LSD (Grof & Dytrych, 1965) |  |  |  |
| MAOI: Iproniazid | Irreversible non-selective MAO inhibitor (Entzeroth & Ratty, 2017) | – Did not alter LSD effects (DeMaar et al., 1960) |  |  | ↓ Milder DMT effects, no hallucinations or disruptions in time and space orientation (Sai Halasz, 1963) |
| Other ADs: Azacyclonol | — | – No reduction of LSD effects (H. Isbell & Logan, 1957)  – No evidence of LSD blocking effects (Clark, 1956) |  | ↓ Attenuated/blocked the effects of mescaline, including hallucinations (Fabing, 1955)  – No impact on mescalines' effects (Clark, 1956)* |  |
| **RECREATIONAL DRUGS**  Alcohol | Potentiates the effects of GABA, inhibits glutamate (Costardi et al., 2015) | – Alcohol's effects diminished by LSD in 87% participants; no change in the effects of LSD (Barrett et al., 2000) | – Subjective effects of alcohol are antagonised by psilocybin in 60% participants; no change in psilocybin’s effects in 80% patients (Barrett et al., 2000) |  |  |
| MDMA | Strong inhibitor of CYP2D6 (O’Mathúna et al., 2008) | – Acute subjective effects lasted longer but no change in the quality (Straumann et al., 2023) |  |  |  |
| **OTHER**  Reserpine | Binds to the storage vesicles of catecholamines (Cheung & Parmar, 2023)  Potent CYP2C19 and CYP2D6 inhibitor (Englund et al., 2014)  P-gp inhibitor (Englund et al., 2014) | ↑ Pretreatment enhanced neurological and psychological LSD effects (Resnick et al., 1965).  ↑ Pretreatment intensified LSD effects of nervousness (H. Isbell & Logan, 1957).  ↑ Pretreatment intensified "toxic" LSD effects (including tremors) and increased duration (Freedman, 1963) |  |  |  |
| Ketanserin | 5-HT_2A_ antagonist (Becker et al., 2023) | ↓ Blocked subjective LSD effects (Preller et al., 2018).  ↓ Less intense subjective LSD effects (Olbrich et al., 2021).  ↓ Blocked subjective LSD effects (Holze et al., 2021).  ↓ Blocked subjective LSD action (Becker et al., 2023). | ↓ Blocked visual perception distortion, depersonalisation and derealisation effects in a dose-dependent manner (Vollenweider et al., 1998) |  |  |
| Ergotamine | 5-HT_1A_ and 5-HT_2A_ agonist (Pokorny et al., 2016) |  | – No significant alterations on subjective experiences (Pokorny et al., 2016) |  |  |
| Niacin | — | ↓ Pretreatment delayed the onset of LSD-effects and prevented most of the perceptual changes from occurring & administration post-LSD ingestion attenuated all effects of LSD within 5 minutes (Agnew & Hoffer, 1955) |  |  |  |
| Scopolamine | Muscarinic cholinergic receptor antagonist (Drevets et al., 2013) | – Did not alter LSD effects (Harris Isbell, 1959) |  |  |  |
| Phenoxybenzamine | Nonselective, irreversible antagonist of the α-adrenergic receptors (Yoham & Casadesus, 2022) | – Did not alter LSD effects (Harris Isbell, 1959) |  |  |  |
| 2C-O (2,4,5-trimethoxyphenethylamine) | — |  |  | ↑ Pretreatment potentiated the effects of mescaline (Dittrich, 1971)* |  |
| Racemic pindolol | β-adrenoceptor antagonist and 5-HT_1A_ mixed agonist-antagonist (Artigas et al., 2001) |  |  |  | ↑ Intensified DMT subjective effects (Strassman, 1996) |
| Methysergide | 5-HT_1_ receptor agonist (Sai-Halasz, 1962) |  |  |  | ↑ Intensified DMT effects, heightened subjective effects and increased hallucinations (Sai-Halasz, 1962) |
| β-carbolines | Potent (reversible) inhibitors of MAO-A (Brito-da-Costa et al., 2020) |  |  |  | ✕ Serious adverse event (hospitalisation) when using with 5-MeO-DMT (Brush et al., 2004)  ✕ Serious adverse event (death) when using with 5-MeO-DMT (Sklerov et al., 2005) |

Main and most relevant molecular targets are brought out, therefore some receptors may be missed, such as those where the drug has low affinity. NA - noradrenaline, 5-HT - serotonin, D - dopamine, * - case report, ↑ - increased/potentiated effects of the psychedelic, ↓ - decreased/blocked effects of the psychedelic, ⇅ - mixed effects on the psychedelic, – - no effects on the psychedelic, ✕ - notable adverse drug event.

### Text S3. Summary of each study.

#### LSD

A total of 23 studies and nine case series/reports were identified, describing interactions with antipsychotics, mood stabilisers, various antidepressants, recreational drugs and other substances. Two studies reported no effects when concomitant drugs were used.

**Antipsychotics: Chlorpromazine**

A couple of studies have been done to investigate the effects of chlorpromazine pretreatment on LSD response. In a series of blocking experiments in which participants were administered 50-100 mg chlorpromazine followed by 40-60 μg of LSD ingestion, the LSD-induced subjective effects were reduced in all doses of chlorpromazine, including anxiety, changes in sensory perception and hallucination (H. Isbell & Logan, 1957). In the reverse experiment whereby LSD was ingested prior to oral administration of chlorpromazine, no significant difference to LSD effects were observed when compared to placebo control. However, intramuscularly-administered chlorpromazine did significantly reduce the intensity of LSD reaction (H. Isbell & Logan, 1957). Murphree (1962) reported contrasting results whereby pretreatment with 25 mg oral chlorpromazine just 30 minutes before was enough to block LSD-induced effects.

On the other hand, Abramson et al. (1960) reported that oral ingestion of chlorpromazine enhanced the physiological and perceptual state, as well as the hallucinations and consciousness induced by LSD, regardless of whether chlorpromazine was ingested 1.5 hours before or after LSD administration. Similarly, in a cohort of schizophrenic patients, Schwarz (1967) reported that chlorpromazine administration after LSD enhanced the effects of LSD such that one patient had thought that he had been given another dose of LSD while another subject reported feeling worse and anxious. Notably, simultaneous ingestion of chlorpromazine with LSD did not alter any of the subjective or physical effects of LSD effects (Abramson et al., 1960; Murphree, 1962).

**Mood stabilisers: Lithium**

A study led by Bonson and Murphy (1996) examined the effects of lithium on LSD-effects. Three participants who had been chronically taking either lithium alone at 600 mg/day or 1000 mg/day; or in combination with the tricyclic antidepressant imipramine at 175 mg/day for 7-50 weeks stated they experienced overall enhanced effects of LSD. Additionally, there was an earlier onset of the effects along with potentiated hallucinatory and psychological effects of LSD. Each of the three participants who were interviewed reported experiencing hallucinations that were more visually vivid or intense than with the normal hallucinogenic effects of LSD which had been expected (Bonson & Murphy, 1996).

**Antidepressants (SSRI and SARI): Fluoxetine, paroxetine, sertraline and trazodone**

Strassman (1992) reported a case of one adult male receiving fluoxetine to treat dysthymia. The subject had markedly decreased sensitivity to LSD whereby the usual dose that was capable of eliciting a full hallucinogenic effect was only ½ or ⅓ as effective in producing the desired effects after pretreatment with fluoxetine (Strassman, 1992). In 1996, a cohort study was conducted by Bonson et al. (1996) to investigate the effects of various SSRIs including fluoxetine, sertraline, paroxetine and serotonin receptor antagonists and reuptake inhibitors (SARI) trazodone, on the LSD response. In general, pretreatment of at least 2 weeks with SSRIs or SARI attenuated the overall response to LSD such as decreased physical, hallucinogenic and psychological effects of LSD regardless of dose or duration of pretreatment with the SSRI. Notably, one subject who had been on fluoxetine for 1 week reported attenuated effects of LSD and after a month of fluoxetine discontinuation, the normal responses to LSD returned (Bonson et al., 1996).

**Antidepressants (TCA): Imipramine, desipramine and clomipramine**

The effects of multiple tricyclic antidepressants including imipramine, desipramine and clomipramine on LSD response were investigated by Bonson and Murphy (1996). Participants were treated with 125 mg to 200 mg/day tricyclic antidepressants for at least 8 weeks prior to administration of LSD at a minimum dose of 80 μg. The pretreatment of the TCAS led to an earlier onset of LSD effects as well as increased the physical, hallucinatory and psychological effects of LSD. Notably, one patient who had stopped desipramine treatment for 12 weeks still experienced the potentiated effects of LSD in terms of psychological and hallucinatory response but not at 20 weeks after. Conversely, another patient who had stopped clomipramine treatment for 12 weeks no longer felt the enhanced effects of LSD (Bonson & Murphy, 1996).

**Antidepressants (MAOI): Isocarboxazid, phenelzine, nialamide and iproniazid**

Three independent studies have investigated the effects of MAOI on LSD response. Resnick et al. (1964) were amongst the first to investigate the effects of MAOI isocarboxazid (brand name Marplan). Pretreatment of participants with 30 mg/day isocarboxazid for 5 days or 2 weeks prior to oral administration of 40 μg LSD attenuated autonomic and psychological responses of LSD (Resnick et al., 1964). Similarly, Bonson and Murphy (1996) reported that pretreatment of participants with 60-75 mg/day phenelzine for 12 weeks followed by 150 μg of LSD dose nearly abolished subjective responses to LSD (Bonson & Murphy, 1996). Pretreatment with nialamide, a different MAOI, daily for 3 weeks to reach a total dose of 3500 mg also blocked the effects of LSD and patients tolerated very high doses of LSD at 500 μg without any clinical or psychotic symptoms (Grof & Dytrych, 1965). In contrast, iproniazid at 100 mg, when taken together with varying doses of LSD, did not alter any physical or subjective effects of LSD (DeMaar et al., 1960).

**Recreational drugs: Alcohol**

There are very limited studies on the effects of alcohol on LSD response. A retrospective self-report study conducted by Barrett et al. (2000) aimed to explore the effects of alcohol and LSD on each other when consumed together. A total of 13 out of 15 participants (86.7%) reported complete blockage of subjective alcohol effects while under the influence of LSD and the remainder reported diminished effect of alcohol. These participants reported that higher doses of alcohol were required to become intoxicated when under the influence of LSD. One of them also noticed that none of the alcohol effects could be felt until the effects of LSD had worn off and if LSD was taken after feeling intoxicated, the alcohol effects would dissipate after LSD intake. In regards to the effects of alcohol on LSD however, none of the participants observed any consequence by alcohol on the subjective effects of LSD (Barrett et al., 2000).

**Recreational drugs: 3,4-methylenedioxymethamphetamine (MDMA)**

Straumann and colleagues (2023) conducted a study to investigate the effects of combining LSD and MDMA in comparison to using each substance separately. Their findings revealed that the combination of LSD (100 μg) and MDMA (100 mg) did not produce significant differences in the subjective experiences reported by participants, as assessed using various instruments such as visual analog scales (VASs), the 5 Dimensions of Altered States of Consciousness (5D-ASC), the Mystical Effects Questionnaire (MEQ), and the Adjective Mood Rating Scale (AMRS), when compared to the effects of LSD used alone.

However, the combination did result in the subjective sensation of "any drug effects" lasting approximately 1.5 hours longer on average compared to LSD alone. MDMA appeared to influence the way LSD was metabolised, leading to higher peak plasma concentrations and a prolonged elimination half-life when both substances were taken together. On a physiological level, the LSD + MDMA combination induced greater increases in blood pressure, heart rate and pupil size when contrasted with LSD alone (Straumann et al., 2023).

**Other: Reserpine**

Reserpine was found to enhance some aspects of LSD-effects in a few studies (Freedman, 1963; H. Isbell & Logan, 1957; Resnick et al., 1965), with a dose-dependent effect observed in one study. Isbell and Logan (1957) first investigated the effects of reserpine in twelve non-psychotic adults in a series of experiments with varying concentrations of reserpine and administration route. Participants pretreated with either a single dose of 1 mg oral reserpine 2 hours prior to 60 μg LSD; 5 mg oral reserpine divided over 2 doses at 10 and 2 hours prior to 60 μg LSD treatment did not experience any blocking effects from reserpine. However, participants treated with 7.5 mg oral reserpine divided over three doses at 22, 10 and 2 hours prior to LSD treatment experienced worsened effects (H. Isbell & Logan, 1957).

Leaving aside the expected reserpine side-effects such as nasal stuffiness, nausea, diarrhoea, vomiting, lethargy, weakness and dizziness, individuals also had increased nervousness and confusion when used LSD together with reserpine. Over half of the patients who received 6 mg of reserpine intramuscular with 60 μg/kg of LSD had gross tremors at rest. Such tremors did not occur when the same dose of LSD was taken alone in these patients (H. Isbell & Logan, 1957).

When participants were pretreated with a lower dose of 6 mg reserpine in total divided over three doses in the same interval via intramuscular injection prior to 60 μg LSD, they experienced increased irritability and mood depression as well as enhanced neurological changes (H. Isbell & Logan, 1957). Interestingly, the combination of LSD and reserpine was reported to cause a specific type of hallucination called "jets" or "jet propulsion." This hallucination is characterised by a hissing sound starting from the back of the head and culminating in a flash of light, along with a sensation of flying or being hurled through the air. This hallucination has not been observed when LSD or reserpine is taken alone. Notably, participants that had the same dose of reserpine via intramuscular injection against a lower dose of LSD at 0.5 μg/kg experienced more positive subjective effects, as reflected in the increased number of LSD-related positive answers and clinical grade, without gross tremors (H. Isbell & Logan, 1957).

Freedman (1963) conducted a study of 14 adult schizophrenic women where the participants were given a single dose of 10 mg reserpine, 2 days prior to 120 μg LSD on test day. All of the participants described that the effects lasted longer and were overall more unpleasant than the control situation without reserpine pretreatment. The experimenters also observed that most of the participants experienced extended periods of tremor and akathisia and in one case, suffered an oculogyric crisis (Freedman, 1963).

Finally, Resnick et al. (1965) examined the effects in three healthy adult males who were treated with 0.5 mg/day of reserpine for two weeks before ingesting 75 μg of LSD. They found that pretreatment with reserpine markedly enhanced the effects of LSD on autonomic and neurologic responses as well as psychological reaction to LSD. Autonomic responses such as blood pressure, heart rate and pupillary size were enhanced and neurologic functions such as cerebellar functions and deep tendon and periosteal reflexes were affected. The psychological reaction to LSD was also enhanced, as indicated by interview, behavioural observation and a symptom rating scale (Resnick et al., 1965).

**Other: Azacyclonol and scopolamine**

Fabing (1955) conducted a number of experiments to investigate the effect of azacyclonol (brand name Frenquel) on LSD-induced responses. Participants were pretreated with 10-30 mg/day of oral azacyclonol for 1 week and observed that the hallucinatory, conscious and mood-altering effects of LSD were blocked but some small physiological effects such as muscle twitching and nausea remained. They followed up and reported that similar blocking effects were also observed when the participants were pretreated with four doses of 50 mg azacyclonol from 50 hours before the test (Fabing, 1955). They then sought to determine whether the blocking effects of azacyclonol persist after treatment had stopped. The two participants in that experiment reported normal LSD-experiences. One of the participants was administered 40 mg azacyclonol intravenously at the fifth hour post-LSD ingestion and felt less anxious while he realised that he was ‘back in the real world’ again and was able to follow conversations more easily (Fabing, 1955). An independent study by Isbell and Logan (1957) reported no changes in LSD-induced effects were observed in participants treated with 20 mg of azacyclonol 3 times a day for 1 week. A reversal experiment whereby intravenous administration of azacyclonol of 100 mg in divided doses after LSD did not alter the intensity of LSD-reactions (H. Isbell & Logan, 1957). Similarly, Clark (1956) did not observe any blocking of LSD-effects when the participants were pretreated with Frequel varying from 10 to 40 mg, four times daily for 2 to 5 days. Azacyclonol, when administered intravenously at the height of LSD reaction, did not alter the experience or the duration of effects (Clark, 1956).

The effects of another antidepressant scopolamine were also investigated whereby various doses of scopolamine were subcutaneously administered simultaneously with 1 μg/kg LSD (H. Isbell et al., 1959). The side effects of scopolamine including pupil dilation, dry mouth and blurred vision increased in a dose-dependent manner and some were more prominent in combination with LSD than LSD alone. Assessment of the mental effects of scopolamine was more complicated. Even though there were an increased number of positive answers after the combination of LSD and scopolamine when compared to LSD alone, the specific responses linked to the positive answers, such as sleepiness and vision blurring, were attributed to scopolamine use alone. The frequency of LSD-specific reactions such as visual perception distortion and optical hallucination were no different in LSD and scopolamine combination when compared to LSD alone (H. Isbell et al., 1959).

**Other: Ketanserin**

Few recent studies have investigated the effects of serotonin receptor antagonist ketanserin on the autonomic nervous system following LSD administration. Preller et al. (2018) conducted a study in 24 healthy participants, whereby participants were pretreated with 40 mg oral ketanserin at three different doses two weeks apart with the final dose 60 minutes before treatment with LSD. Neuroimaging scans performed at 75 and 300 minutes after treatment administration showed that ketanserin blocked the global brain connectivity induced by LSD. Notably, the global brain connectivity in somatomotor network is correlated with subjective effects of LSD (Preller et al., 2018). In a double-blind placebo controlled trial, ketanserin pretreatment followed by LSD administration led to a significant shift towards the parasympathetic activity when compared to placebo only or placebo-LSD controls (Olbrich et al., 2021). The shift in autonomic nervous system was also associated with less intense subjective experience of LSD measured by the Five Dimensional Altered States of Consciousness (5D-ASC) scale (Olbrich et al., 2021). Another independent study by Holze et al. (2021) reported that ketanserin pretreatment blocked the subjective effects of a high dose of 200 μg LSD to levels similar to 25 μg of LSD. The scores for “Anxious Ego- Dissolution” and “Oceanic Boundlessness” as well as any subjective “good” and “bad” drug effects at 200 μg LSD + Ketanserin were similar to 25 μg of LSD. They also observed that the combination significantly lowered the blood pressure, body temperature and heart rate of the participants to similar levels as in placebo controls (Holze et al., 2021). The blocking effects of ketanserin on LSD were also corroborated by Becker et al. (2023) who observed that the participants pretreated with 40 mg ketanserin followed by 100 μg of LSD one hour later, experienced less LSD-induced auditory and visual alterations as well as ego-dissolution and emotional excitement. However, ketanserin pretreatment did not significantly alter overall mystical experiences or plasma brain-derived neurotrophic factor (BDNF) levels induced by LSD. Ketanserin-pretreated participants also experienced shortened duration of LSD-effects from 8.5 to 3.5 hours without any change in pharmacokinetics of LSD (Becker et al., 2023).

**Other: Niacin**

Agnew and Hoffer (1955) examined the effects of niacin (vitamin B3) on the effects of LSD. Ten healthy adults were administered 200 mg niacin intravenously post LSD administration. Attenuation of LSD effects were observed in all participants and niacin injection markedly diminished the proprioceptive, perceptual and cognitive effects of LSD. However, when participants were pretreated with 3 grams of oral niacin daily for 3 days followed by 100 μg of LSD on test day, the onset of LSD effects was delayed and participants still experienced feelings of unreality and depersonalisation, but without LSD-induced perceptual changes (Agnew & Hoffer, 1955).

**Other: Phenoxybenzamine**

Isbell et al. (1959) examined the blocking effects of varying doses of phenoxybenzamine pretreatment at 0.5 mg, 1 mg and 1.0 mg/kg that were administered 2 hours prior to 1 μg/kg LSD. None of the doses of phenoxybenzamine significantly altered LSD-induced effects including pupil dilation, pulse rate, blood pressure, knee reflex or subjective positive feelings (H. Isbell et al., 1959).

**Additional case report**

Additionally, there is a report about a 16 year old individual who consumed two doses of LSD while being on fluoxetine (20 mg/day) treatment and developed marked stupor hours after LSD ingestion, followed by focal seizure that progressed to a grand mal convulsion (Picker et al., 1992). However, this report is omitted from our results as the person previously ingested LSD 30 times during fluoxetine treatment without such adverse effects and it is unclear whether the person had any previous experiences with the same batch of LSD.

#### Psilocybin

A total of ten studies and one case report were identified, describing interactions with anxiolytics, antipsychotics, antidepressants, recreational drugs and other substances.

**Anxiolytics: Buspirone**

A study conducted by Pokorny et al. (2016) pretreated 19 healthy participants with 20 mg buspirone one hour before the oral ingestion of 0.17 mg/kg psilocybin. Buspirone pretreatment markedly reduced the psilocybin-induced visual hallucinations and to a lesser extent, facilitated imagination but did not affect audio-visual synaesthesia. Additionally, it attenuated the psilocybin-induced loosening of ego-boundaries, changes in sense of time and euphoria (Pokorny et al., 2016).

**Antipsychotics: Chlorpromazine**

In one study by Keeler (1967), eight healthy volunteers were given either 50 mg of chlorpromazine or placebo two hours before oral administration of 0.2 mg/kg psilocybin. They reported that the participants pretreatment with chlorpromazine experienced significantly decreased psilocybin-induced pupil dilation, visual perception distortion and after-image response (Keeler, 1967).

**Antipsychotics: Risperidone and haloperidol**

In a randomised, placebo-controlled trial conducted by Vollenweider et al. (1998), it was reported that both risperidone and haloperidol reduced the effects of psilocybin. Risperidone affected psilocybin in a dose-dependent manner, with 0.5 mg blocking the effects of psilocybin by 69-78% and 1 mg completely abolishing psilocybin-induced psychosis (98-99%). Haloperidol pretreatment diminished the feelings of oceanic boundlessness and derealization, without affecting visual perception distortions or hallucinations. However, participants who underwent haloperidol treatment experienced increased anxiety and dread of ego-dissolution (Vollenweider et al., 1998).

**Antidepressant: Escitalopram**

Becker et al. (2022) conducted a randomised placebo-controlled trial involving 23 healthy participants with two experimental test sessions to examine the interaction effects between the antidepressant escitalopram and psilocybin. Briefly, participants were pretreated with 10 mg escitalopram daily for 7 days, followed by 20 mg/day for another 7 days including test day which was ingested 2 hours before 25 mg psilocybin administration. They observed that pretreatment with escitalopram significantly reduced psilocybin-induced ego disintegration and anxiety, but had no effect on depersonalisation, oceanic boundlessness or euphoria. There were also no significant differences between the groups in overall mystical experiences induced by psilocybin although it was able to reduce acute adverse effects on blood pressure. Notably, escitalopram did not normalise psilocybin-induced increase in plasma BDNF (Becker et al., 2022).

**Other: Ketanserin**

In a separate experiment, Vollenweider et al. (1998) pretreated 25 healthy volunteers with 20 mg of oral ketanserin or 40 mg of placebo, followed by 0.25 mg/kg psilocybin 75 minutes later. It was reported that ketanserin blocked psilocybin-induced psychosis in a dose-dependent manner. Pretreatment with 20 mg of ketanserin reduced the altered state of consciousness induced by psilocybin by 50-70%, while 40 mg of ketanserin completely prevented the effects of psilocybin in 4 out of 5 participants. In the remaining participant, the effects of psilocybin were markedly reduced by 75-87% (Vollenweider et al., 1998). Similarly, Carter et al. (2005) pretreated 8 healthy participants with 50 mg oral ketanserin 90 minutes prior to 0.215 mg/kg psilocybin and examined the effects on attentional tracking ability and spatial working memory as well as subjective effects. Ketanserin significantly blocked the subjective effects of psilocybin including “Oceanic Boundlessness” (depersonalisation, euphoria), “Anxious Ego Dissolution” (ego-disintegration, anxiety) and “Visual Restructuralization” (hallucinations, synaesthesia) but not “Auditory alterations” or “Reduction of Vigilance” which measures acoustic hallucinations and reduced alertness respectively. However, ketanserin pretreatment did not rescue the psilocybin-induced decrease on attentional tracking ability, whereby participants were to track specific coloured disk on the monitor along with other coloured disks, the disks were then all coloured the same during the prompt and the user has to select the correct disk of a target colour (Carter et al., 2005). They followed up with another experiment (Carter et al., 2007) with the same experimental design but examined the effects on binocular rivalry whereby participants were presented with two incongruent images which typically results in perceptual alternation between the two images. Pretreatment with ketanserin did not alter the psilocybin-induced slowing of binocular rivalry. However, ketanserin pretreatment has consistently lowered the psilocybin-induced effects on Oceanic Boundlessness, Anxious Ego Dissolution and Visual Restructuralization scores without affecting the “Reduction of Vigilance” score as well as the arousal score which is a new addition to a more recent version of 5D-ASC (Carter et al., 2007).

**Other: Ergotamine**

Pokorny et al. (2016) conducted a study whereby 17 participants were pretreated with 3 mg ergotamine 100 minutes before the oral administration of 0.17 mg/kg. They observed that ergotamine pretreatment did not significantly alter any of the psilocybin-induced effects including sense of euphoria, alteration to sense of time, anxious ego disintegration, or visual hallucinations based on the 5D-ASC score (Pokorny et al., 2016).

**Recreational drugs: Alcohol**

Barrett et al. (2000) conducted a retrospective self-report study to examine the effects of non-intoxicating levels of alcohol on psilocybin when consumed either before or after psychedelic ingestion. Fifteen participants underwent structured interviews consisting of a series of standardised open-ended questions: “Have you ever used alcohol while under the influence of psychedelic or psychedelic while under the influence of alcohol?”, “Did you notice any difference in the effect of either the alcohol or psychedelic when you used the two together?”,“What was the nature of the difference?”. The answers were rated on a five-point Likert scale by the investigators. The subjective effects of alcohol are partially antagonised by psilocybin whereby users reported diminished response to alcohol but none of the users reported complete blocking of alcohol effects. However, 80% of the participants did not experience any difference in effects of psilocybin when consumed together with alcohol (Barrett et al., 2000).

#### Mescaline

A total of one study and three case series/reports were identified, describing interactions with antipsychotics, antidepressants and other compounds.

**Antipsychotics: Chlorpromazine**

In one study, 25 schizophrenic patients received either mescaline by intravenous injection or oral LSD, followed by chlorpromazine or promazine intravenous injection (Lesse, 1958). The authors noted that the anxiety caused by mescaline and LSD administration was abolished in 68% (17 out of 25) of the participants by chlorpromazine or promazine. No details were given on the number of patients who received mescaline or LSD or on the doses and timing of administration.

**Antidepressants: Azacyclonol**

Another study investigated azacyclonol (Frenquel) as a blocking agent for mescaline effects (Fabing, 1955). They used four healthy patients, two of whom received pretreatment with 50 mg azacyclonol at 50, 38, 26 and 2 hours before ingesting 400 mg of mescaline sulphate, while the other two received placebo pretreatment and 100 mg intravenous injections of azacyclonol at 4.5 and 5 hours, respectively. The patients who received azacyclonol as pretreatment developed nausea and minor subjective effects (such as feeling tension in leg muscles and disruptions in thought continuity), but did not experience hallucinations or other psychic accompaniments. One of the other two patients experienced euphoria and sensations of levitation with brightened colours, while the other two developed somatic hallucinations and extreme coldness (vomited at the 4^th^ hour). After receiving a 100 mg intravenous treatment of azacyclonol, the effects of mescaline were blocked, and both patients returned to “normal” within an hour.

**Case reports**

A self-experiment was conducted by Clark (1956), who took azacyclonol four times a day for three days before ingesting 200 mg and 400 mg of mescaline sulphate. He reported that the intoxications were identical in all respects to those he previously experienced after taking the same doses without azacyclonol pretreatment, and did not experience any blocking effect of azacyclonol (Clark, 1956).

Another self-experiment conducted by Dittrich (1971), who used mescaline in combination with 2,4,5-trimethoxyphenethylamine (2C-O). He designed a blind experiment in which he first ingested 300 mg of 2C-O, then 100 mg and 200 mg of 2C-O with mescaline, and finally 250 mg of mescaline alone. He did not experience any subjective effects while taking 2C-O alone. In regards to the experiment with pretreatment, he observed differences in two tests. Mescaline was shown to decrease performance in a test measuring concentration on optical details, while pretreatment with 2C-O resulted in even greater deterioration of performance than mescaline alone. The time to complete a test (semantic differential) was measured and increased under mescaline compared to placebo and even further after pretreatment with 2C-O (Dittrich, 1971).

#### DMT and ayahuasca

A total of three studies were identified for DMT, describing interactions with antidepressants as well as other substances. One case report was found for ayahuasca.

**Antidepressants (MAOI): Iproniazid**

In an experimental study by Sai Halasz (1963), seven volunteers (four males and three females aged between 21-36 years) were pre-treated with the MAOI iproniazid for four days, followed by a two-day washout period to eliminate the direct effect of iproniazid while preserving its MAOI activity. On the fifth day, two individuals received a reduced dose of DMT (0.35-0.55 mg/kg), while the other five received a dose of 0.65-0.83 mg/kg.

The individuals who received the reduced dose of DMT reported no significant changes in their perception of time, space, or sensory experiences. However, they reported an "odd" or "strange" feeling, which they found difficult to describe. The five volunteers who received the higher DMT dose experienced two distinct phases of effects. The first phase lasted 14-24 minutes and was similar to the effects of DMT, albeit less pronounced. They reported seeing a few hallucinations, which lacked vivid colours and only occurred with their eyes closed. In the second phase, the hallucinations disappeared and their perception of time and space returned to baseline levels. However, the feeling of oddness remained and even intensified (Sai Halasz, 1963).

**Other: racemic pindolol and methysergide**

In a four-cell double-blind randomised study by Strassman (1996), twelve volunteers received a sub-hallucinogenic dose (0.1 mg/kg) of intravenous DMT or saline placebo, in combination with either 30 mg of oral racemic pindolol or placebo-pindolol. The results of the study showed that pindolol (a potent 5-HT_1A_ antagonist) pre-treatment enhanced DMT effects by two to three times, which was substantiated by scores on the hallucinogen rating scale. Four to six clinical clusters (affect, volition, somatic effects, perception, cognition and intensity) demonstrated a significant enhancement by pindolol. Heart rate responses were blunted, probably due to pindolol's anti-sympathetic effects, while mean arterial blood pressure effects were enhanced. Prolactin responses were reduced, while those of adrenocorticotropin were unaffected (Strassman, 1996).

In a quasi-experimental study by Sai-Halasz (1962), 40 patients (20 males and 20 females aged between 18-44 years) received a DMT dose of 0.76-1.03 mg/kg. Two to three months later, 15 of those individuals participated in the second experiment. Seven of them were administered the same dose as the first time (0.81-0.89 mg/kg DMT), while eight patients had their dose reduced to 50-80% of their first dose. In the second experiment, the participants were pre-treated with methysergide (1-methyllysergic acid butanolamide), a compound structurally similar to LSD and a serotonergic antagonist but without psychic effects. Methysergide was administered perorally (1-2 mg) 30-40 min before DMT or intramuscularly (0.5 mg) 10 min before DMT. Out of the seven patients who received the same DMT dose as the first time, five individuals reported very intense aggravation of DMT subjective effects, such as more intense hallucinations, more brilliant colours, deeper loss of time and perception and lost all relation with the world, unlike the first time. The other two individuals reported heightened subjective effects of DMT, but not to such a marked degree. Four individuals who took 65-80% less DMT than their first time experienced similar effects as their first experience. The four individuals who took 50-60% of their first-time dose did not experience a more pronounced hallucinatory state than their first experience. In one case, the effects were even less pronounced (Sai-Halasz, 1962).

**Case reports**

There was one case report by Callaway and Grob (1998) of a 36-year-old male individual who was receiving fluoxetine treatment (20 mg/day) and who participated in a ceremonial ayahuasca session. Approximately one hour after ingesting about 100 ml of ayahuasca, the patient developed sweating, shivering, tremors and confusion, which gradually progressed and led to severe nausea, vomiting and disorientation. The patient did not receive any treatment and the symptoms subsided after four hours following the ingestion of ayahuasca (Callaway & Grob, 1998).

Additionally, another case report by Bakim et al. (2012) involved a 42 year old male who received a combination of quetiapine at a daily dose of 1000 mg and fluoxetine at a daily dose of 40 mg. While the individual did not consume ayahuasca and therefore not included in the main results, he did ingest a spoonful of harmal (*Peganum harmala*) to treat his haemorrhoids, a plant used in ayahuasca brew. Two hours after ingestion of harmal the individual developed symptoms such as nausea, vomiting, sweating, tremors, followed by confusion with visual hallucinations, agitation and loss of orientation and was diagnosed with serotonin syndrome. Symptoms resolved within 24 hours after starting the treatment with cyproheptadine, chlorpromazine and diazepam (Bakim et al., 2012).

#### 5-MeO-DMT

Two case reports involving the use of 5-MeO-DMT were found reporting serious adverse events.

**Case reports**

One case report (Brush et al., 2004) described a 17-year old male who ingested an extract from three Syrian rue (*Peganum harmala*) seeds, smoked 10 mg of 5-MeO-DMT and snorted an additional 15-20 mg of 5-MeO-DMT. This led to severe agitation, hallucinations, vomiting, diaphoretic skin as well as high body temperature (40.7 °C) and increased heart rate (186 bpm) as measured in the emergency department after hospitalisation. Urine analysis confirmed the presence of harmaline and harmine.

The second case report (Sklerov et al., 2005) described a 25-year-old man who went camping with family and friends in a national park, where he consumed "herbal tonics" and subsequently died in his sleep. Investigation revealed he had ingested a preparation from South American tree bark called "ooasca," followed by ingestion of tryptamines about four hours later. The autopsy did not reveal any anatomical cause of death. However, toxicological analysis of the heart blood showed the presence of several substances, including DMT, 5-MeO-DMT, tetrahydroharmine, harmaline and harmine. Urine analysis also showed traces of diphenhydramine. The medical examiner determined that the cause of death resulted from intoxication caused by hallucinogenic amines, with the manner of death remaining undetermined.

#### References

Abramson, H. A., Rolo, A., & Stache, J. (1960). Lysergic Acid Diethylamide (LSD-25) Antagonists- Chlorpromazine.pdf. *Journal of Neuropsychiatry 1*, 307–310.

Agnew, N., & Hoffer, A. (1955). Nicotinic acid modified lysergic acid diethylamide psychosis. *The Journal of Mental Science*, *101*(422), 12–27.

Artigas, F., Celada, P., Laruelle, M., & Adell, A. (2001). How does pindolol improve antidepressant action? *Trends in Pharmacological Sciences*, *22*(5), 224–228.

Bakim, B., Sertcelik, S., & Tankaya, O. (2012). A Case of Serotonin Syndrome with Antidepressant Treatment and Concomitant use of The Herbal Remedy (Peganum Harmala). *Klinik Psikofarmakoloji Bülteni-Bulletin of Clinical Psychopharmacology*, *22*(4), 359–361.

Barrett, S. P., Archambault, J., Engelberg, M. J., & Pihl, R. O. (2000). Hallucinogenic drugs attenuate the subjective response to alcohol in humans. *Human Psychopharmacology*, *15*(7), 559–565.

Becker, A. M., Holze, F., Grandinetti, T., Klaiber, A., Toedtli, V. E., Kolaczynska, K. E., Duthaler, U., Varghese, N., Eckert, A., Grünblatt, E., & Liechti, M. E. (2022). Acute Effects of Psilocybin After Escitalopram or Placebo Pretreatment in a Randomized, Double-Blind, Placebo-Controlled, Crossover Study in Healthy Subjects. *Clinical Pharmacology and Therapeutics*, *111*(4), 886–895.

Becker, A. M., Klaiber, A., Holze, F., Istampoulouoglou, I., Duthaler, U., Varghese, N., Eckert, A., & Liechti, M. E. (2023). Ketanserin Reverses the Acute Response to LSD in a Randomized, Double-Blind, Placebo-Controlled, Crossover Study in Healthy Participants. *The International Journal of Neuropsychopharmacology / Official Scientific Journal of the Collegium Internationale Neuropsychopharmacologicum* , *26*(2), 97–106.

Bonson, K. R., Buckholtz, J. W., & Murphy, D. L. (1996). Chronic administration of serotonergic antidepressants attenuates the subjective effects of LSD in humans. *Neuropsychopharmacology: Official Publication of the American College of Neuropsychopharmacology*, *14*(6), 425–436.

Bonson, K. R., & Murphy, D. L. (1996). Alterations in responses to LSD in humans associated with chronic administration of tricyclic antidepressants, monoamine oxidase inhibitors or lithium. *Behavioural Brain Research*, *73*(1–2), 229–233.

Boyd-Kimball, D., Gonczy, K., Lewis, B., Mason, T., Siliko, N., & Wolfe, J. (2019). Classics in Chemical Neuroscience: Chlorpromazine. *ACS Chemical Neuroscience*, *10*(1), 79–88.

Brito-da-Costa, A. M., Dias-da-Silva, D., Gomes, N. G. M., Dinis-Oliveira, R. J., & Madureira-Carvalho, Á. (2020). Toxicokinetics and Toxicodynamics of Ayahuasca Alkaloids N,N-Dimethyltryptamine (DMT), Harmine, Harmaline and Tetrahydroharmine: Clinical and Forensic Impact. *Pharmaceuticals* , *13*(11). https://doi.org/10.3390/ph13110334

Brush, D. E., Bird, S. B., & Boyer, E. W. (2004). Monoamine oxidase inhibitor poisoning resulting from Internet misinformation on illicit substances. *Journal of Toxicology. Clinical Toxicology*, *42*(2), 191–195.

Callaway, J. C., & Grob, C. S. (1998). Ayahuasca preparations and serotonin reuptake inhibitors: a potential combination for severe adverse interactions. *Journal of Psychoactive Drugs*, *30*(4), 367–369.

Carter, O. L., Burr, D. C., Pettigrew, J. D., Wallis, G. M., Hasler, F., & Vollenweider, F. X. (2005). Using psilocybin to investigate the relationship between attention, working memory, and the serotonin 1A and 2A receptors. *Journal of Cognitive Neuroscience*, *17*(10), 1497–1508.

Carter, O. L., Hasler, F., Pettigrew, J. D., Wallis, G. M., Liu, G. B., & Vollenweider, F. X. (2007). Psilocybin links binocular rivalry switch rate to attention and subjective arousal levels in humans. *Psychopharmacology*, *195*(3), 415–424.

Cheung, M., & Parmar, M. (2023). *Reserpine*. StatPearls Publishing.

Clark, L. D. (1956). Further studies of the psychological effects of frenquel and a critical review of previous reports. *The Journal of Nervous and Mental Disease*, *123*(6), 557–560.

Costardi, J. V. V., Nampo, R. A. T., Silva, G. L., Ribeiro, M. A. F., Stella, H. J., Stella, M. B., & Malheiros, S. V. P. (2015). A review on alcohol: from the central action mechanism to chemical dependency. *Revista Da Associacao Medica Brasileira*, *61*(4), 381–387.

DeMaar, E. W. J., Williams, H. L., Miller, A. I., & Pfeiffer, C. C. (1960). Effects in man of single and combined oral doses of reserpine, iproniazid, andD-lysergic acid diethylamide. *Clinical Pharmacology and Therapeutics*, *1*(1), 23–30.

Dittrich, A. (1971). Alteration of behavioural changes induced by 3,4,5-trimethoxyphenylethylamine (mescaline) by pretreatment with 2,4,5-trimethoxyphenylethylamine. *Psychopharmacologia*, *21*(3), 229–237.

Drevets, W. C., Zarate, C. A., Jr, & Furey, M. L. (2013). Antidepressant effects of the muscarinic cholinergic receptor antagonist scopolamine: a review. *Biological Psychiatry*, *73*(12), 1156–1163.

Englund, G., Lundquist, P., Skogastierna, C., Johansson, J., Hoogstraate, J., Afzelius, L., Andersson, T. B., & Projean, D. (2014). Cytochrome p450 inhibitory properties of common efflux transporter inhibitors. *Drug Metabolism and Disposition: The Biological Fate of Chemicals*, *42*(3), 441–447.

Entzeroth, M., & Ratty, A. K. (2017). Monoamine oxidase inhibitors—revisiting a therapeutic principle. *Open Journal of Depression*, *06*(02), 31–68.

Fabing, H. D. (1955). Frenquel, a blocking agent against experimental LSD-25 and mescaline psychosis; preliminary note on its clinical application. *Neurology*, *5*(5), 319–328.

Fagiolini, A., Comandini, A., Catena Dell’Osso, M., & Kasper, S. (2012). Rediscovering trazodone for the treatment of major depressive disorder. *CNS Drugs*, *26*(12), 1033–1049.

Freedman, D. X. (1963). Psychotomimetic drugs and brain biogenic amines. *The American Journal of Psychiatry*, *119*, 843–850.

Gillman, P. K. (2007). Tricyclic antidepressant pharmacology and therapeutic drug interactions updated. *British Journal of Pharmacology*, *151*(6), 737–748.

Grof, S., & Dytrych, Z. (1965). Blocking of LSD reaction by premedication with Niamid. *Activitas Nervosa Superior*, *7*(3), 306.

Holze, F., Vizeli, P., Ley, L., Müller, F., Dolder, P., Stocker, M., Duthaler, U., Varghese, N., Eckert, A., Borgwardt, S., & Liechti, M. E. (2021). Acute dose-dependent effects of lysergic acid diethylamide in a double-blind placebo-controlled study in healthy subjects. *Neuropsychopharmacology: Official Publication of the American College of Neuropsychopharmacology*, *46*(3), 537–544.

Isbell, H., & Logan, C. R. (1957). Studies on the diethylamide of lysergic acid (LSD-25). II. Effects of chlorpromazine, azacyclonol, and reserpine on the intensity of the LSD-reaction. *A.M.A. Archives of Neurology and Psychiatry*, *77*(4), 350–358.

Isbell, H., Logan, C. R., & Miner, E. J. (1959). Studies on lysergic acid diethylamide (LSD-25). III. Attempts to attenuate the LSD-reaction in man by pretreatment with neurohumoral blocking agents. *A.M.A. Archives of Neurology and Psychiatry*, *81*(1), 20–27.

Isbell, Harris. (1959). Effects of Various Drugs on the LSD Reaction. *Psychopharmacology Frontiers*, 361–364.

Keeler, M. H. (1967). Chlorpromazine Antagonism of Psilocybin Effect. *International Journal of Neuropsychiatry*.

Lesse, S. (1958). Psychodynamic relationships between the degree of anxiety and other clinical symptoms. *The Journal of Nervous and Mental Disease*, *127*(2), 124–130.

Loane, C., & Politis, M. (2012). Buspirone: what is it all about? *Brain Research*, *1461*, 111–118.

Massot, O., Rousselle, J. C., Fillion, M. P., Januel, D., Plantefol, M., & Fillion, G. (1999). 5-HT1B receptors: a novel target for lithium. Possible involvement in mood disorders. *Neuropsychopharmacology: Official Publication of the American College of Neuropsychopharmacology*, *21*(4), 530–541.

Murphree, H. B. (1962). Quantitative studies in humans on the antagonism of lysergic acid diethylamide by chlorpromazine and phenoxybenzamine. *Clinical Pharmacology and Therapeutics*, *3*, 314–320.

Noehr-Jensen, L., Zwisler, S. T., Larsen, F., Sindrup, S. H., Damkier, P., & Brosen, K. (2009). Escitalopram is a weak inhibitor of the CYP2D6-catalyzed O-demethylation of (+)-tramadol but does not reduce the hypoalgesic effect in experimental pain. *Clinical Pharmacology and Therapeutics*, *86*(6), 626–633.

Olbrich, S., Preller, K. H., & Vollenweider, F. X. (2021). LSD and ketanserin and their impact on the human autonomic nervous system. *Psychophysiology*, *58*(6), e13822.

O’Mathúna, B., Farré, M., Rostami-Hodjegan, A., Yang, J., Cuyàs, E., Torrens, M., Pardo, R., Abanades, S., Maluf, S., Tucker, G. T., & de la Torre, R. (2008). The consequences of 3,4-methylenedioxymethamphetamine induced CYP2D6 inhibition in humans. *Journal of Clinical Psychopharmacology*, *28*(5), 523–529.

Peng, L., Gu, L., Li, B., & Hertz, L. (2014). Fluoxetine and all other SSRIs are 5-HT2B Agonists - Importance for their Therapeutic Effects. *Current Neuropharmacology*, *12*(4), 365–379.

Picker, W., Lerman, A., & Hajal, F. (1992). Potential interaction of LSD and fluoxetine. *The American Journal of Psychiatry*, *149*(6), 843–844.

Pokorny, T., Preller, K. H., Kraehenmann, R., & Vollenweider, F. X. (2016). Modulatory effect of the 5-HT1A agonist buspirone and the mixed non-hallucinogenic 5-HT1A/2A agonist ergotamine on psilocybin-induced psychedelic experience. *European Neuropsychopharmacology: The Journal of the European College of Neuropsychopharmacology*, *26*(4), 756–766.

Preller, K. H., Burt, J. B., Ji, J. L., Schleifer, C. H., Adkinson, B. D., Stämpfli, P., Seifritz, E., Repovs, G., Krystal, J. H., Murray, J. D., Vollenweider, F. X., & Anticevic, A. (2018). Changes in global and thalamic brain connectivity in LSD-induced altered states of consciousness are attributable to the 5-HT2A receptor. *ELife*, *7*. https://doi.org/10.7554/eLife.35082

Resnick, O., Krus, D. M., & Raskin, M. (1964). LSD-25 action in normal subjects treated with a monoamine oxidase inhibitor. *Life Sciences*, *3*, 1207–1214.

Resnick, O., Krus, D. M., & Raskin, M. (1965). Accentuation of the psychological effects of LSD-25 in normal subjects treated with reserpine. *Life Sciences*, *4*(14), 1433–1437.

Sai Halasz, A. (1963). The effect of MAO inhibition on the experimental psychosis induced by dimethyltryptamine. *Psychopharmacologia*, *4*, 385–388.

Sai-Halasz, A. (1962). The effect of antiserotonin on the experimental psychosis induced by dimethyltryptamine. *Experientia*, *18*(18), 137–138.

Schwarz, C. J. (1967). Paradoxical Responses to Chlorpromazine after LSD.pdf. *Psychosomatics*, *8*(4), 210–211.

Sklerov, J., Levine, B., Moore, K. A., King, T., & Fowler, D. (2005). A fatal intoxication following the ingestion of 5-methoxy-N,N-dimethyltryptamine in an ayahuasca preparation. *Journal of Analytical Toxicology*, *29*(8), 838–841.

Sproule, B. A., Otton, S. V., Cheung, S. W., Zhong, X. H., Romach, M. K., & Sellers, E. M. (1997). CYP2D6 inhibition in patients treated with sertraline. *Journal of Clinical Psychopharmacology*, *17*(2), 102–106.

Strassman, R. J. (1992). Human hallucinogen interactions with drugs affecting serotonergic neurotransmission. *Neuropsychopharmacology: Official Publication of the American College of Neuropsychopharmacology*, *7*(3), 241–243.

Strassman, R. J. (1996). Human psychopharmacology of N,N-dimethyltryptamine. *Behavioural Brain Research*, *73*(1–2), 121–124.

Straumann, I., Ley, L., Holze, F., Becker, A. M., Klaiber, A., Wey, K., Duthaler, U., Varghese, N., Eckert, A., & Liechti, M. E. (2023). Acute effects of MDMA and LSD co-administration in a double-blind placebo-controlled study in healthy participants. *Neuropsychopharmacology: Official Publication of the American College of Neuropsychopharmacology*. https://doi.org/10.1038/s41386-023-01609-0

Vollenweider, F. X., Vollenweider-Scherpenhuyzen, M. F., Bäbler, A., Vogel, H., & Hell, D. (1998). Psilocybin induces schizophrenia-like psychosis in humans via a serotonin-2 agonist action. *Neuroreport*, *9*(17), 3897–3902.

Yoham, A. L., & Casadesus, D. (2022). *Phenoxybenzamine*. StatPearls Publishing.
